# A Robotic High-Throughput Grid-Search Platform for Mapping Phase Behavior in Triblock Copolymer–Homopolymer Blends

**DOI:** 10.1021/acsnano.6c01299

**Published:** 2026-06-09

**Authors:** Saroj Upreti, Lan Xu, Md Moniruzzaman, Yunfei Wang, Kailash Adhikari, Sabin Baral, Derek Patton, Boran Ma, Jie Xu, Ruipeng Li, Chenhui Zhu, Wenjie Xia, Xiaodan Gu

**Affiliations:** † School of Polymer Science and Engineering, Center for Optoelectronic Materials and Devices, 5104 University of Southern Mississippi, Hattiesburg, Mississippi 39406, United States; ‡ Department of Aerospace Engineering, 1177 Iowa State University, Ames, Iowa 50011, United States; § Advanced Light Source, 1666 Lawrence Berkeley National Laboratory, Berkeley, California 94720, United States; ∥ Nanoscience and Technology Division, 1291 Argonne National Laboratory, Lemont, Illinois 60439, United States; ⊥ National Synchrotron Light Source II (NSLS-II), Brookhaven National Lab, Upton, New York 11973, United States; # Pritzker School of Molecular Engineering, The University of Chicago, Chicago, Illinois 60637, United States

**Keywords:** high-throughput processing, phase behavior, order–disorder transition, nanostructure morphology, triblock copolymer thin films

## Abstract

We present a high-throughput experimental investigation of the phase behavior in triblock copolymers (PS-*b*-PB-*b*-PS and PS-*b*-PI-*b*-PS) and polystyrene (PS) homopolymer blends as a function of homopolymer molecular weight (MW) and blend ratio. Using a robotic thin-film processing platform (NOVA) integrated with Grazing Incidence Small-Angle X-ray Scattering (GISAXS) and Atomic Force Microscopy (AFM), we systematically mapped the order–disorder transition (ODT) boundaries and domain spacing evolution across a broad MW range (4.0–101.3 kDa) with varying homopolymer loadings (10% to 90%). The results reveal three distinct regimes: low-MW homopolymers, corresponding to the wet-brush regime produced only gradual domain swelling before disordering at high blend ratios (weight fraction); medium-MW homopolymers, corresponding to thedry-brush regime induced significant domain spacing increase up to 80% followed by earlier disordering, while high-MW homopolymers led to macrophase separation with minimal changes in domain spacing. Additionally, coarse-grained molecular dynamics simulations confirmed our experimental finding that in the low-MW region, the PS homopolymer uniformly distributed in the PS domain. These findings demonstrate that homopolymer molecular weight critically governs both the extent of domain swelling and the onset of disorder in triblock copolymer systems. This high-throughput platform enables the rapid mapping of composition–morphology relationships and can be integrated with AI/ML tools for designing next-generation nanostructured polymers.

## Graphical abstract

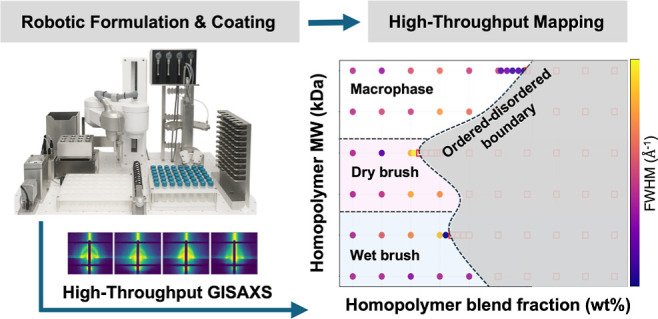

## Introduction

The self-assembly behavior of block copolymers (BCPs), which enables diverse nanoscale morphologies, makes them ideal candidates for advanced applications such as nanofiltration membranes,^1,2^ nanolithography templates,^3,4^ photonic crystals,^5,6^ and drug delivery systems.^7,8^ Since BCPs comprise two or more chemically distinct polymer blocks covalently linked together, the incompatibility between these blocks primarily drives microphase separation.^9^ In bulk or solution, the equilibrium morphologies predicted by mean-field methods such as self-consistent field theory (SCFT) depend on the Flory–Huggins interaction parameter (χ), degree of polymerization (*N*), and block volume fraction (*f*).^10,11^ In thin films, additional complexity arises from interfacial energies and confinement effects, which therefore require control of surface energy to achieve well-ordered oriented domains.^12–14^ A substantial body of knowledge has been accumulated on the self-assembly behavior of diblock copolymers over the past three decades.

Blending homopolymers (HPs) with BCPs offers a powerful and versatile strategy to tailor copolymer self-assembly behavior, eliminating the laborious need to synthesize new polymers. Both theoretical and experimental studies have demonstrated that such blending enables control over domain sizes, stabilizes complex or metastable morphologies, and modulates order–disorder transition (ODT) characteristics.^15–18^ Within the theoretical framework, foundational SCFT analyses have shown that the molecular weight (MW) of HPs plays a pivotal role in dictating phase behavior, domain dimensions, and ODT characteristics.^19,20^ This occurs because the addition of HPs often alters the segregation strength and domain composition, while also shifting the balance between osmotic pressure (favor swelling) and the entropic penalty associated with stretching the BCP chains (favor contraction).^19,21^ In particular, the ratio of HP molecular weight (MW) to that of the corresponding block defines distinct regimes. The wet-brush regime (MW ratio ≪ 1) leads to uniform HP distribution and predictable domain swelling; the dry-brush regime (MW ratio ≈1) results in preferential segregation toward domain centers, enhancing swelling and potentially altering internal structures, whereas the macrophase-separation regime (MW ratio ≫ 1) leads to two phases between BCP and HPs.^22–24^

Several simulation studies on diblock BCP systems have collectively advanced understanding of micelle formation, morphological transitions, and the stabilization of complex phases.^25–27^ Greenall et al. studied poly­(styrene–butadiene) diblock copolymer–polystyrene blends using SCFT to calculate micelle core radii, corona thicknesses, and critical micelle concentrations.^25^ Ly et al. explored BCP with HP under 3D spherical confinement using computational modeling, showing that the MW and confinement size of HP significantly influence self-assembly and nanostructure formation.^26^ Martínez-Veracoechea et al. used Monte Carlo simulations on BCP systems to identify conditions under which bicontinuous phases form, providing insights for designing materials with high surface areas and enhanced transport properties.^27^

Experimental studies on such BCP/HP blends have further established that incorporating HPs provides a powerful way of tuning nanoscale morphologies, ordering kinetics, and phase transitions.^28–35^ Early work by Koizumi et al. mapped the interplay between macro- and microphase transitions in bulk BCP/HP mixtures, demonstrating how HP content influences the ordering process.^28^ Similarly, Orso and Green showed that HP addition increases domain dimensions and alters morphology as a function of blend composition.^29^ Subsequent studies expanded on these findings: Choi et al. observed that blending with HP and BCP can induce diverse, coexisting morphologies.^30^ Moreover, Sun et al. demonstrated that HP’s MW critically affects the stacking behavior of perforated lamellar structures, enabling controlled transitions between AB and ABC stacking arrangements.^31^ Other work has shown how MW and composition control specific morphological outcomes. Doerk et al. demonstrated that blending symmetric PS-*b*-PMMA with low-MW PS and PMMA in the wet-brush regime promotes uniform HP distribution, resulting in significantly faster ordering kinetics and an order-of-magnitude increase in grain size compared to neat BCPs.^32^ Spontak et al. showed that blending symmetric PS-*b*-PI with low-MW PS (<20 kDa) at intermediate MW BCP (30–80 kDa) promotes an ordered bicontinuous double-diamond (OBDD) structure near 66 vol % styrene, yielding stable bicontinuous morphologies with tunable domain size. They also found that very low (20–30 kDa) or very high (>120 kDa) BCP MW shift the system toward lamellar, cylindrical, or disordered phases.^33^ Other studies have also demonstrated clear increase or decrease in domain spacing depending on the MW and concentration of the HPs used.^34,35^ For instance, Peng et al. reported that blending 10–20 wt % of low-MW PMMA (15.0 kDa) with symmetric PS-*b*-PMMA (263.0 kDa) systematically increased domain spacing from 63 to 66 nm, whereas high-MW PMMA (102.6 kDa) produced a contraction to 61 nm at 10 wt % and an expansion to 70 nm at 20 wt %.^34^ A study by Toth et al. using symmetric PS-*b*-PMMA (75 kDa) with an inherent domain spacing of 38.5 nm showed that blended with low-MW PS (1 kDa) and PMMA­(1 kDa) HPs at a 30% mass fraction decreased the spacing to 36.5 nm. In contrast, blending with high-MW PS (22 kDa) and PMMA (22 kDa) increased the domain spacing to 44.5 nm.^35^

Triblock BCP systems represent an advancement over diblock BCP systems because of the presence of an additional block and greater synthetic constraints. Theoretical, experimental, and simulation studies have demonstrated that triblock BCPs can self-assemble into a wide range of nanostructures, including complex bicontinuous network phases that are less accessible in diblock BCP systems.^36,37^ Going one step further, blending triblock BCP with HPs enables additional tuning knobs over nanostructure and interfacial properties, with outcomes strongly dependent on polymer architecture, MW, and blend composition. Chen et al. used SCFT and demonstrated that adding C-type HPs to ABC triblock BCP confined within nanopores enables the formation of tunable helical nanostructures, where the number of helical strands (1–6) is controlled by the HP’s chain length, concentration, and pore diameter.^38^ Furthermore, Xie and Shi used SCFT and random-phase approximation to show that ABA and BAB triblock copolymers (with identical chemical composition but different topologies) displayed different phase behaviors when blended with A-type HPs. Specifically, ABA/A blends stabilize Frank–Kasper phases, while BAB/A blends exhibit poorer miscibility, highlighting the critical role of topology in phase design.^39^ Additionally, Monte Carlo simulations by Huang et al. demonstrated that in ABA/A and ABA/B blend films, increasing the HP volume fraction or the molecular mass ratio enhances macrophase separation, and triblock BCP/HP blends showed more irregular morphologies compared to the diblock BCP counterpart.^40^ Currently, only a handful of experimental studies have explored the effect of HP’s MW on the blending behavior of HPs with triblock BCPs. Additional experimental studies are needed to substantiate the existing theoretical predictions.

To overcome the challenge of limited experimental capability, robot-based high-throughput processing and characterization of polymeric materials can be leveraged. High-throughput approaches have recently enabled the simultaneous exploration of multiple synthesis, processing, and compositional variables to efficiently map structure–property relationships and to develop advanced materials such as organic photovoltaics, supramolecular cages, and functional biomaterials.^41–44^ Representative robotic platforms in the polymer processing field include the PolyBot platform at Argonne National Lab, the Autonomous Formulation Laboratory (AFL) at NIST, and the Polymer Analysis and Discovery Array (PANDA) at Boston University.^45–47^ While PolyBot specializes in autonomous polymer electronic discovery, AFL integrates automated fluid handling with Small Angle X-ray Scattering (SAXS) to map complex phase spaces.^45,46^ Further, PANDA provides an open-source architecture for high-throughput electrodeposition and functional characterization of polymer films through systematic combination of fluid handling, electrochemistry, and optical measurements.^47^ These platforms altogether transform the traditionally labor-intensive workflows into high-throughput, automation-enabled rapid mapping of structure–property relationships previously inaccessible through manual experimentations.

This work focuses on leveraging high-throughput robotic processing to fabricate polymeric blend films, followed by high-throughput morphological characterization using grazing incidence small-angle X-ray scattering (GISAXS) to study the phase behavior of triblock BCP/HP blends. By implementing a systematic, grid-based approach, we performed a comprehensive study on the effect of PS HP’s MW on the morphological behavior of polystyrene-block-polybutadiene-block-polystyrene (PS-*b*-PB-*b*-PS) and polystyrene-block-polyisoprene-block-polystyrene (PS-*b*-PI-*b*-PS) triblock BCP thin films. More specifically, this study systematically explores how variations in HP’s MW shift the free-energy landscape leading to the order–disorder transition (ODT) by altering the entropy of mixing, enthalpic selectivity, and effective segregation strength (χN). We observed that incorporating low-MW PS HP (4.0 and 8.4 kDa) gradually increased the domain spacing of PS-*b*-PB-*b*-PS films from approximately 38 to 47 nm, while medium-MW PS HP (16.6 and 28.7 kDa) produced a more pronounced expansion, up to 68 nm. In contrast, high-MW PS HP (55.0 and 101.3 kDa) induced macrophase separation with only minimal changes in microdomain spacing. This work further advances the understanding of phase behavior in binary blend systems of HP and triblock BCP thin film, thereby contributing to the design of next-generation copolymer materials.

## Results and Discussion

### High-Throughput Workflow for Triblock BCP/HP Blend Thin Films

In this work, we developed a high-throughput workflow to efficiently prepare and characterize hundreds of triblock BCP/HP blend thin-film samples, thereby enabling a systematic and effective study of their phase behavior. The overall workflow, encompassing high-throughput processing, characterization, and data analysis, is illustrated in Figure 1. The sample preparation was performed through a custom-designed robotic thin-film processing platform, NOVA.^48^

**Figure 1:**
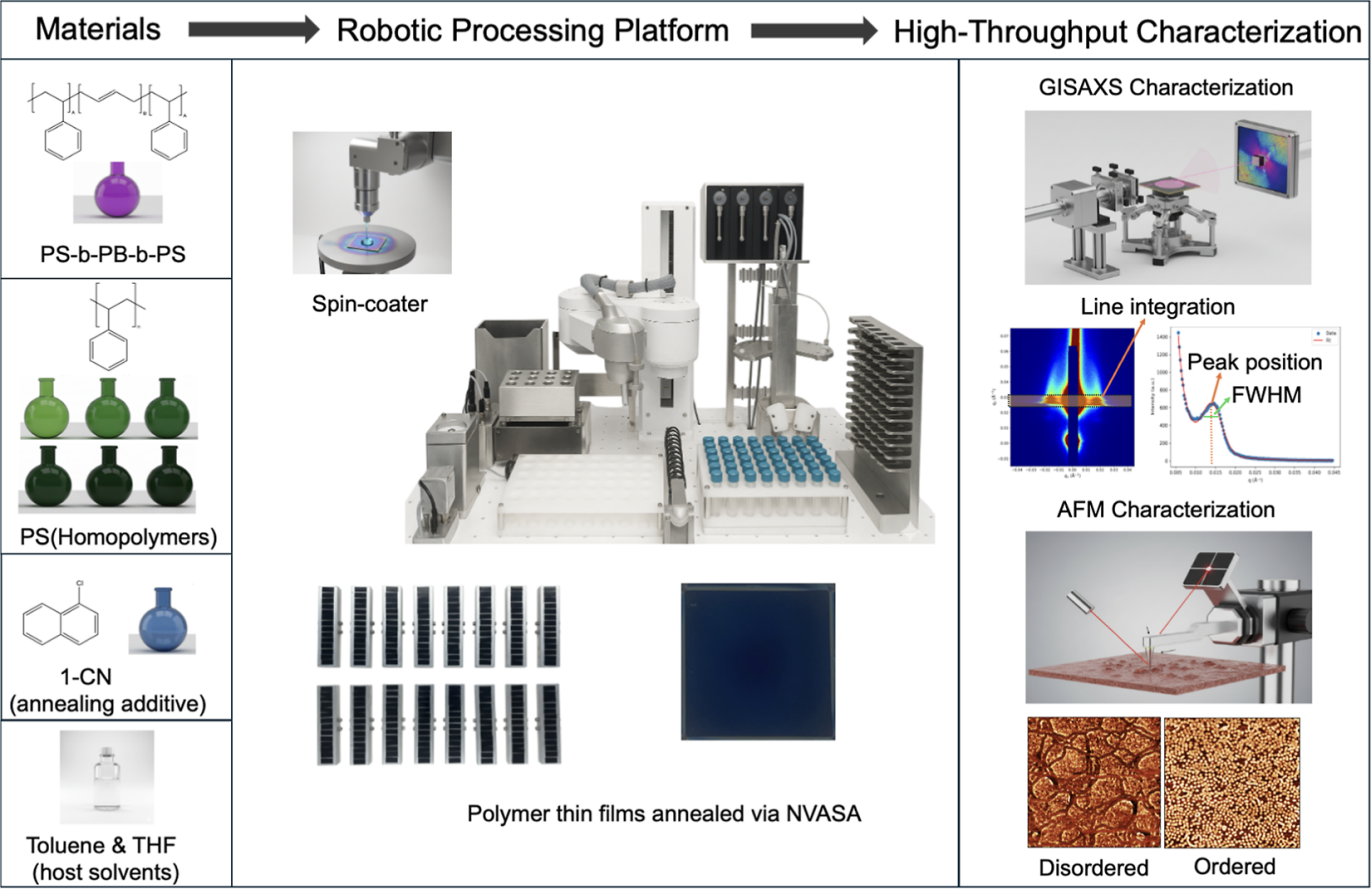
Schematic overview of the high-throughput workflow for robotic sample processing, morphological characterization, and data analysis. (LEFT) Materials used: PS-*b*-PB-*b*-PS triblock BCP (focus), six PS HPs of different molecular weights, the additive annealing solvent 1-chloronaphthalene (1-CN), and host solvents used to dissolve polymer (toluene and THF). (MIDDLE) High-throughput robotic processing platform. (RIGHT) High-throughput morphology characterization using GISAXS and AFM on selected samples; GISAXS data analysis to extract peak position (domain spacing) and full width at half-maximum (FWHM, a measure of ordering) from 1D line profiles obtained by integrating along the Yoneda peak of the 2D GISAXS patterns.

Stock solutions of triblock BCPs, HPs, and additive were processed into thin films using NOVA, capable of automated mixing, stirring, heating, coating, and additive annealing. A demonstration video illustrating the workflow in detail is provided in the Supporting Information (supporting movie, S-Movie). As shown in Figures 2A and S1, NOVA comprises several integrated modules, each responsible for specific processing functions. After the desired input volumes of each stock solution are specified via a Python-based interface, the robotic arm retrieves a vial, places it at the sample loading position, uncaps it, and dispenses the prescribed volumes of each solution through a syringe pump. After the software records the mass of the dispensed solution, the arm recaps the vial, and mechanically stirs it to complete the formulation. Next, the system performs spin coating of the prepared sample formulation. The robot uncaps the vial again, then withdraws a small volume (∼75 μL) of the solution, positions a blank silicon wafer onto the spin coater, dispenses the solution, and spin-coats a film under the desired conditions, in this case, 2000 rpm for 30 s. The entire formulation and coating process requires approximately 3 min per sample. In a typical experimental run, the system can formulate and coat more than 144 thin-film samples in a continuous run and thousands of samples with periodic restocking of sample vials and silicon wafers.

**Figure 2:**
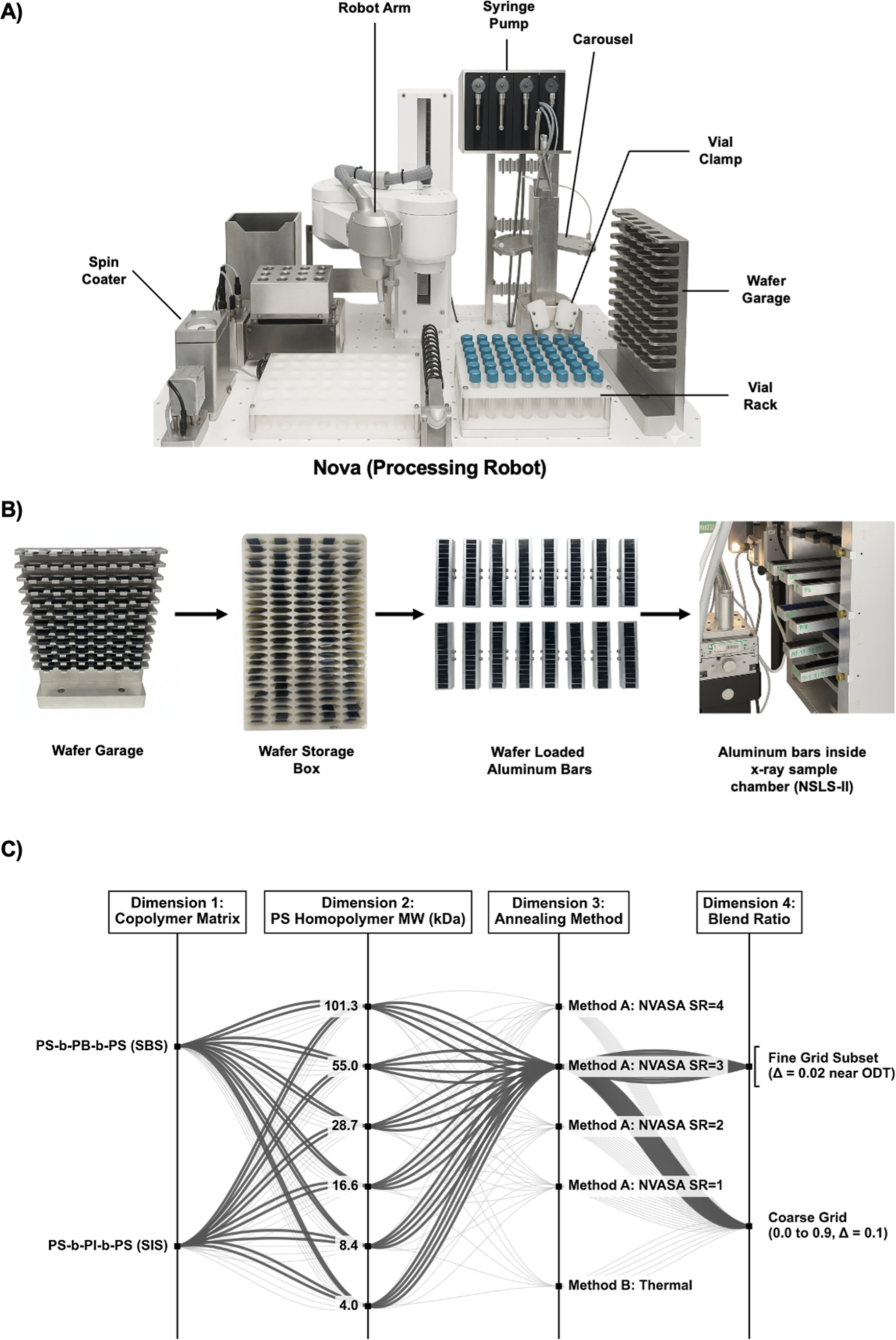
Components of the high-throughput processing and characterization workflow. (A) NOVA, the polymer formulation and coating robot (center), showing its major functional modules. (B) Schematic of the workflow, including the wafer garage containing films coated by NOVA, the high-capacity storage box used for film storage and safe transport, aluminum bars loaded with polymer films, and the sample chamber accommodating 12 aluminum bars for high-throughput GISAXS experiments at the CMS beamline of NSLS-II. (C) 4-dimensional parameter space using parallel axes to show each coordinate. The darker color represents the data in the main article while lighter color represents all other samples (available in Supporting Information).

The coated films from the wafer garage were then unloaded and placed in a custom-made high-capacity storage and transport box. After transportation to the Complex Material Scattering (CMS) at the National Synchrotron Light Source-II (NSLS-II), the films were mounted onto aluminum bars and placed in the sample chamber for GISAXS experiments (Figure 2B). An X-ray measurement throughput of about 12–15 samples per hour was achieved. The rate-limiting step was sample alignment in both the vertical direction and the incidence angle, which can be further optimized. Sample exposure times were on the order of seconds, thanks to the high photon flux from the synchrotron source.

Five different incidence angles were measured ranging from 0.08° to 0.16°. During a typical three day beamtime, around 750 individual samples could be measured, and over 3000 X-ray images collected. This workflow demonstrates the integration of a robotic system for high-throughput processing and scattering of multiple silicon wafer samples, facilitating the rapid collection of large scattering data sets. For GISAXS data analysis, a horizontal line cut integration along the Yoneda peak was carried out, followed by peak fitting of resulting 1D data to extract the peak position and full-width half-maximum (FWHM) values, corresponding to the domain spacing and degree of ordering, respectively. The GISAXS data analysis and peak fitting protocols are described in detail in our previous report.^49^ Overall, the NOVA platform allows much higher throughput in sample formulation and characterization. A comparison and contrast between NOVA and other existing platforms (PolyBot, PANDA, Ada, and AFL) can be found in Table S1.

## Results Overview

In this study, we aim to elucidate the effects of the HP (PS) mixing/blend ratio (BR) and MW on the phase behavior of the triblock BCPs PS-*b*-PB-*b*-PS (15%-*b*-70%-*b*-15%) and PS-*b*-PI-*b*-PS (7.5%-*b*-85%–7.5%). Based on theoretical calculations (Table S2), both triblock BCPs reveal moderate to strong segregation strengths at equilibrium conditions. Using the robotic system NOVA, we prepared over 400 samples to capture a full picture of blend samples’ phase behavior. The thickness of the prepared films roughly ranged from 105 to 120 nm. While the potential influence of thickness variation on phase behavior is acknowledged, a detailed investigation of these effects is beyond the focus of this work. We studied six different MWs of PS, ranging from 4.0 kDa to 101.3 kDa. Each HP was blended with PS-*b*-PB-*b*-PS and PS-*b*-PI-*b*-PS at a 10% increment in HP weight fraction; in other words, the BR ranged from 0.1 to 0.9 in steps of 0.1. While this 10% step size was necessary to efficiently map the macrophase separation boundaries across such a large parameter space, we acknowledge that it limits the observation of complex behaviors at ultralow volume fractions, which remains an area for future high-resolution study. For PS-*b*-PB-*b*-PS triblock BCP, after an initial grid search, three of six HPs were further explored using finer BR increments of 0.02 near the observed order–disorder transition (ODT) to pinpoint the boundary precisely. Based on our previously reported “non-volatile additive solvent annealing” (NVASA), a high BP annealing additive 1-chloronaphthalene (CN) was added to each sample formulation at varying degrees of additive loading to promote ordering.^50^ Initially, samples were prepared at four different swell ratios (SRs) ranging from 1 to 4. These values of SRs were simply calculated as 1 + *m*_a_ */m*_p_, where *m*_a_ and *m*_p_ represented the mass of incorporated additive and the mass of polymer, respectively. Subsequently, a copy of these samples was thermally annealed at 150 °C for 12 h in a glovebox (inert environment), and the results were compared with those from NVASA. A summarized 4D grid-search parameter map, with clear indication of the relevant data and where they can be found (main article or Supporting Information), is shown in Figure 2C.

The morphology of samples was characterized by GISAXS to understand their phase behavior, and representative results are shown in Figure 3. The 2D scattering patterns, example images in Figure 3A and the comprehensive data in Figures S5–S18, were first converted into 1D profiles (Figure 3C) plotted as scattering intensity (*I*) versus scattering vector (*q*).^49^ The FWHM values were used to determine the order–disorder phase boundary. Samples were classified as disordered if they (i) exhibited no detectable peak or (ii) showed a relatively broad FWHM (>0.01 Å^–1^) compared to ordered samples. Those disordered samples were further confirmed by AFM and are discussed in the following section. To note, this manuscript primarily focuses on PS/SBS blend systems when it comes to further AFM- and simulation-related studies, although general trends of PS/SIS blends are also explored.

**Figure 3:**
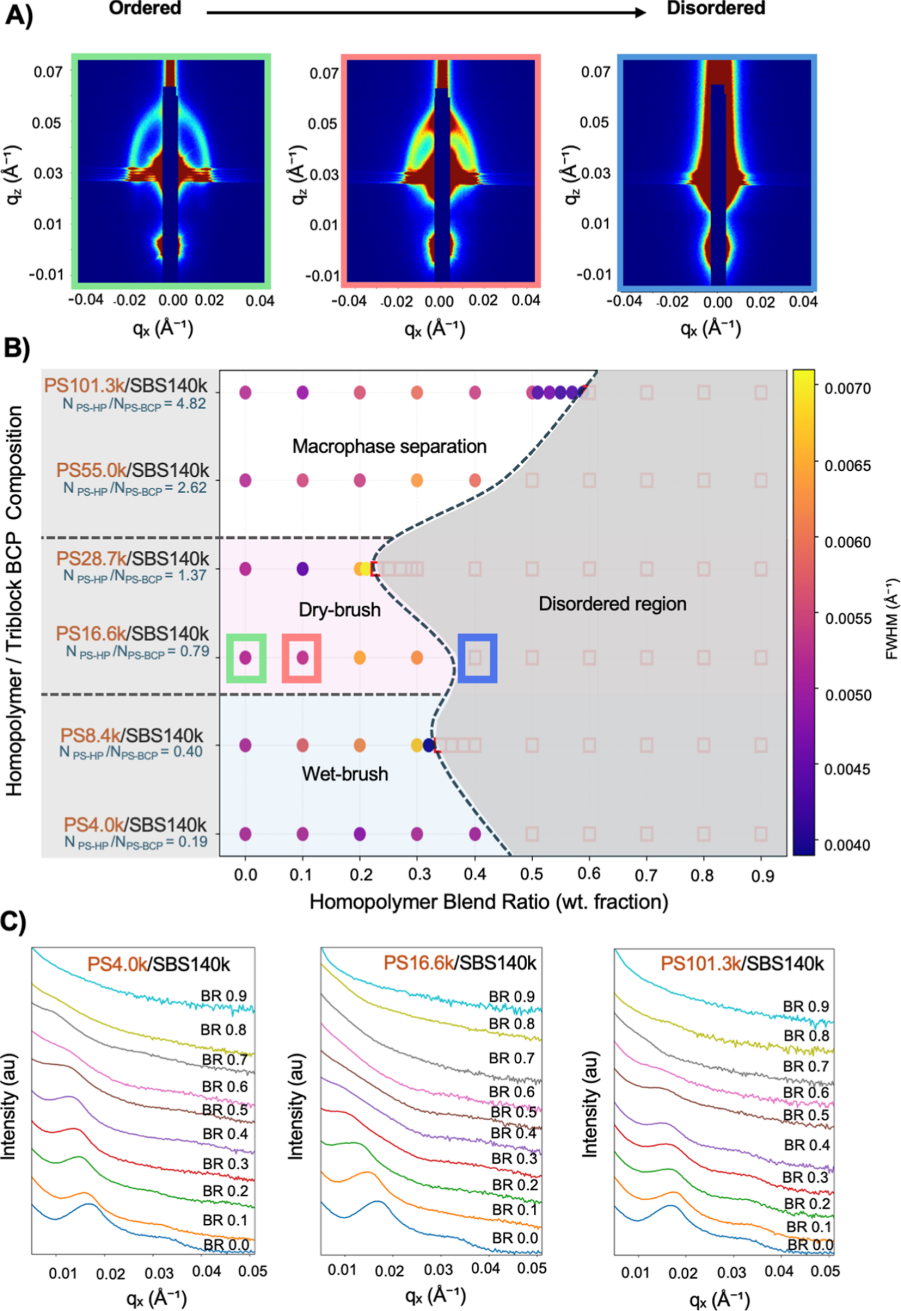
(A) Representative 2D GISAXS scattering patterns from ordered to disordered states. (B) Grid search map of the order–disorder boundary (ODT), determined from fwhm values obtained by fitting the 1D scattering profiles. Within the map, solid circular markers denote blends exhibiting a distinct primary scattering peak (ordered or macrophase-separated regimes). The color of these solid markers directly correlates to the full width at half maximum (FWHM) of the primary peak, as indicated by the right-hand color bar. Faded open squares designate blends in the disordered region that lack a distinct primary scattering peak. The bold green, red, and blue square outlines highlight the specific data points corresponding to the representative 2D scattering patterns shown in panel A. (C) 1D intensity vs q profiles for representative blend systems across the three regimes, with homopolymer blend ratios ranging from 0 to 0.9. All scattering data presented here were processed at a swell ratio of 3 (SR3). Other data at different swell ratios are provided in the Supporting Information.

The ∼30 wt % styrene content of the neat PS-*b*-PB-*b*-PS is expected to enable cylindrical microdomains. Bulk SAXS and baseline GISAXS measurements (Figure S19) confirm an ordered cylindrical domain, with a pronounced primary scattering peak (q*) and a broad higher-order shoulder near $\sqrt{3}q^{*}$ and ${\sqrt{7}q}^{*}$ . Notably, the higher-order scattering features in these rapidly processed thin-film libraries do not reveal strongly. Hence, assigning order–order phase transitions is not highly reliable, although increasing PS homopolymer content is thermodynamically expected to induce such transitions. Accordingly, the high-throughput workflow developed here focuses on the evolution of the primary domain spacing to map the order–disorder transition (ODT) and macrophase-separation boundaries across the full compositional space.

Based on the ratio of the HP PS’s MW to that of the PS block in the triblock BCPs, the triblock BCP/HP blend systems can be categorized into three regimes: wet-brush (MW ratio <0.75), dry-brush (*M*_W_ 0.75–1.5), and macrophase separation (*M*_W_ ratio >2). These empirical thresholds align with classical self-consistent field theory (SCFT) expectations for chain stretching and localization.^22,24^ Our designated cutoffs (<0.75, 0.75–1.5, and >2) represent the empirical boundaries where these distinct structural and chain-stretching behaviors become experimentally dominant across our high-throughput grid. Figure 3B shows the ODT map of the PS-*b*-PB-*b*-PS system obtained under additive annealing at a SR of 3. The films processed at lower SRs exhibited significantly higher FWHM values, indicating that the structures remained partially kinetically trapped with poor long-range order (Figure S20). Increasing the additive concentration to SR3 minimized the FWHM across the blend ratios providing optimal chain mobility to overcome spin-coating kinetic barriers. As SR3 represented the highest order, closer to the equilibrated thermodynamic phase, the ODT maps were created using this SR.

In the wet-brush regime (4.0–8.4 kDa), disorder occurs only at higher BRs (0.3–0.4), as low-MW HPs PS mix uniformly within the PS domains in triblock BCP, leading to gradual swelling and preserving ordering across a broad compositional range. The dry-brush regime (16.6–28.7 kDa) exhibited earlier disorder (BR = 0.2–0.3) due to preferential segregation of the HPs into domain centers, leading to rapid swelling but a narrower stability window.^15^ As established by theoretical models and experimental neutron scattering, this core-localization minimizes severe block stretching penalties; in an ABA triblock, it forces the cylinders apart, strains the continuous matrix bridging chains, and drives early onset of disorder.^19,51^ At the other extreme, the macrophase-separation regime (55.0–101.3 kDa) maintains the order of triblock BCP to higher BRs (>0.5), reflecting poor compatibility between BCP and HPs. Notably, in this region, however, the GISAXS data show peak shifting, pronounced broadening, and eventual loss of long-range order indicating that the HP does not fully segregate during the rapid quench of spin-coating. Because severe diffusion difficulties persisted even at SR3 conditions, the resulting slow dynamics and strong entanglement led to kinetic trapping within or near the BCP domains. This partial trapping leads to slight domain swelling (peak shifting) and substantial defect generation (peak broadening). At higher homopolymer loadings, the severe packing frustration and interfacial disruption suppress long-range microphase separation, driving the BCP into a disordered state.

The ODT map for PS-*b*-PI-*b*-PS (Figure S2) across the three MW-defined regimes exhibited a nonmonotonic dependence of disorder on BR. In the wet-brush limit (4.0 kDa), ordering is lost at BR = 0.1–0.2. The dry-brush cases (8.4 and 16.6 kDa) showed disorder at BR = 0.1–0.2 and 0.3–0.4, respectively, indicating enhanced swelling and a modest shift to higher SR in ODT from 8.4 to 16.6 kDa. Within the macrophase regime, the ODT shifted to lower BRs for 28.7 kDa (0.2–0.3), then to higher BRs for 55.0 kDa (0.4–0.5), and finally returned to intermediate BRs for 101.3 kDa (0.3–0.4). Unlike the SBS system, the SIS ODT boundary exhibits an oscillatory trend. This distinct behavior is possibly due to the high compositional asymmetry of the SIS matrix (15 wt % PS versus 30 wt % for SBS). Low-MW homopolymers (wet-brush) uniformly swell the highly curved native SIS domains, causing rapid structural disruption at low blend ratios. Conversely, medium-MW homopolymers (dry-brush) preferentially segregate to the domain cores. This core-localization relieves local chain stretching without aggressively altering the interfacial curvature, stabilizing the ordered morphology at higher homopolymer loadings. The subsequent boundary oscillations at higher MWs reflect a competition between severe kinetic trapping and the transition into true macrophase separation.

Comparing between thermally annealed and additive annealed data (Figures S3 and S4) for PS/SBS blends, NVASA shifted the ODT to higher HP loadings, with the strongest stabilization observed in the dry-brush regime (16.6–28.7 kDa). Differences are modest in the wet-brush regime, while much earlier transitions are observed in the macrophase-separation region. A notable exception is the PS4.0k/SBS140k blend, which disordered at lower HP loading under additive annealing, showing a shifted ODT to lower BRs likely related to enhanced plasticization that reduced the effective χN in the wet-brush limit. These ODT boundary shifts between thermally annealed and additive annealed blends reflect different ordering pathways: thermal annealing targets the thermodynamic equilibrium of the pure binary polymer blend, while NVASA drives toward a ternary equilibrium determined by a balance of dilution (promoting disorder) and selectivity (promoting order) which can vary with MW of the PS brush (PS/SBS). For example, as swelling stretched the BCP chains in the dry-brush regime, 1-CN solvent possibly moves to the domain interfaces and relieves this chain tension keeping the ordered phase stable. In the macrophase-separation regime, while thermal annealing kinetically traps the highly entangled chains in forcibly mixed, metastable ordered states, the enhanced mobility provided by the 1-CN plasticizer allows the massive homopolymer chains to rapidly cluster and disrupt the block copolymer interfaces, driving the system into a disordered state earlier.

Under NVASA with 1-CN at a SR of 3, PS/SBS blends exhibit a clear MW-dependent evolution of domain spacing. In the wet-brush regime (Figure 4A,B), domain spacing increased gradually and monotonically with BR, increasing from 38 to 47 nm as 30% PS HP is added for both MWs. By contrast, the dry-brush regime (Figure 4C,D) showed a steep, nonlinear increase at modest loadings. Domain spacing increased to 68 nm at a BR of 0.3 for 16.6 kDa and to 60 nm at a BR of 0.2 for 28.7 kDa, beyond which compositions approached the ODT. At high MW (Figure 4E,F), domain spacing remained comparatively stable with BR, 40–43 nm. Park and Sancaktar also reported a related system of PS-*b*-PB-*b*-PS (117.6 kDa, 24% PS) blending with different MWs of minority block PS, although their study did not systematically vary both BR and HP MW.^52^ In their study, the triblock BCP with a domain spacing of 28.4 nm when blended with 10 wt % of PS with MWs of 4.0, 12.0, 17.0, 21.0, and 27.0 kDa resulted in domain spacings of 28.8, 29.5, 36.7, 38.4, and 39.7 nm, respectively. The comparison plots in Figure S21 clearly show, in the referenced work, the domain swelling is gradual in the wet-brush regime followed by a single swift increase before approaching the dry-brush regime, where the swelling is more rapid. We observed a similar trend only at the higher HP loading (20 and 30 wt %), but the rapid domain spacing increasing behavior was not observed with 10 wt % PS loading at the dry-brush regime.

**Figure 4:**
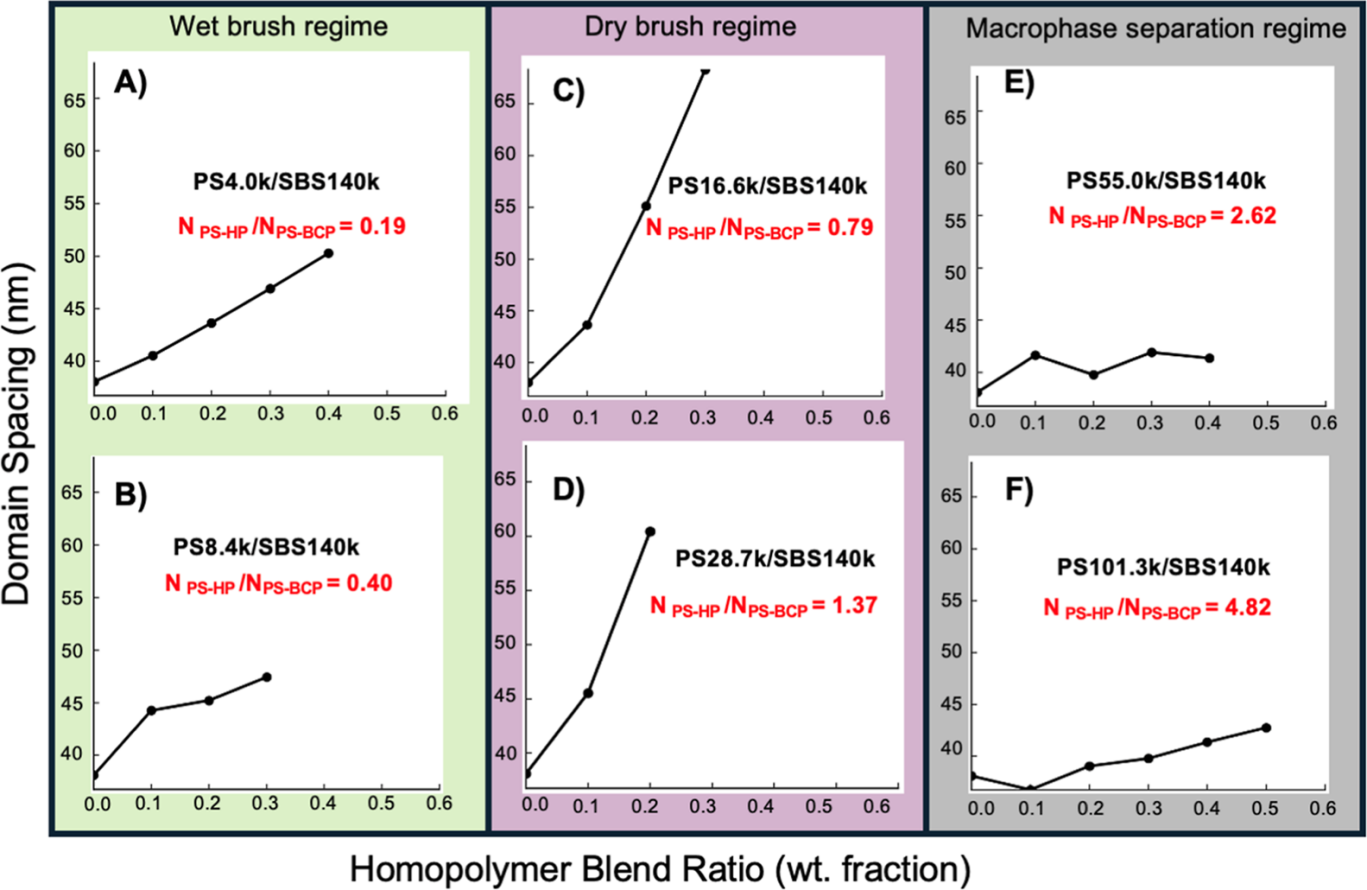
Evolution of domain spacing in PS-*b*-PB-*b*-PS/PS blends as a function of homopolymer molecular weight (MW) and blend ratio (BR). The data reveal three distinct regimes: (A,B) low-MW homopolymers (wet-brush regime) cause gradual domain swelling, (C,D) medium-MW homopolymers (dry-brush regime) produce pronounced and rapid domain expansion, and (E,F) high-MW homopolymers (macrophase-separation regime) lead to macrophase separation with only minimal changes in spacing.

Comparable regime-specific trends are also observed in PS/PS-*b*-PI-*b*-PS blends (Figure 6, Table S3). Since the lower-MW HP systems were disordered with 20 wt % PS loading, a comprehensive trend could be captured with only 10 wt % PS loading. Like the PS/PS-*b*-PB-*b*-PS system, no clear wet-brush/dry-brush boundary was observed at 10 wt % PS loading; the domain spacing increased from 34 nm (neat SIS) to 44 and 48 nm for the ratios of 0.35 and 0.75, respectively. This value plateaued at 47 nm for a ratio of 1.47, which later decreased to 38, 36, and 36 nm for ratios of 2.55, 4.88, and 9.00, respectively. López-Barrón et al. used similar PS-*b*-PI-*b*-PS (82.6 kDa, 14 wt % PS) triblock BCP and blended with 10 wt % PS (3.8 kDa, ratio = 0.65) to observe domain spacing shift from 42 nm (neat triblock BCP) to 55 nm.^53^ This domain swelling rate trend matches closely with the PS8.4k/SIS150k system with a ratio of 0.75.

**Figure 5:**
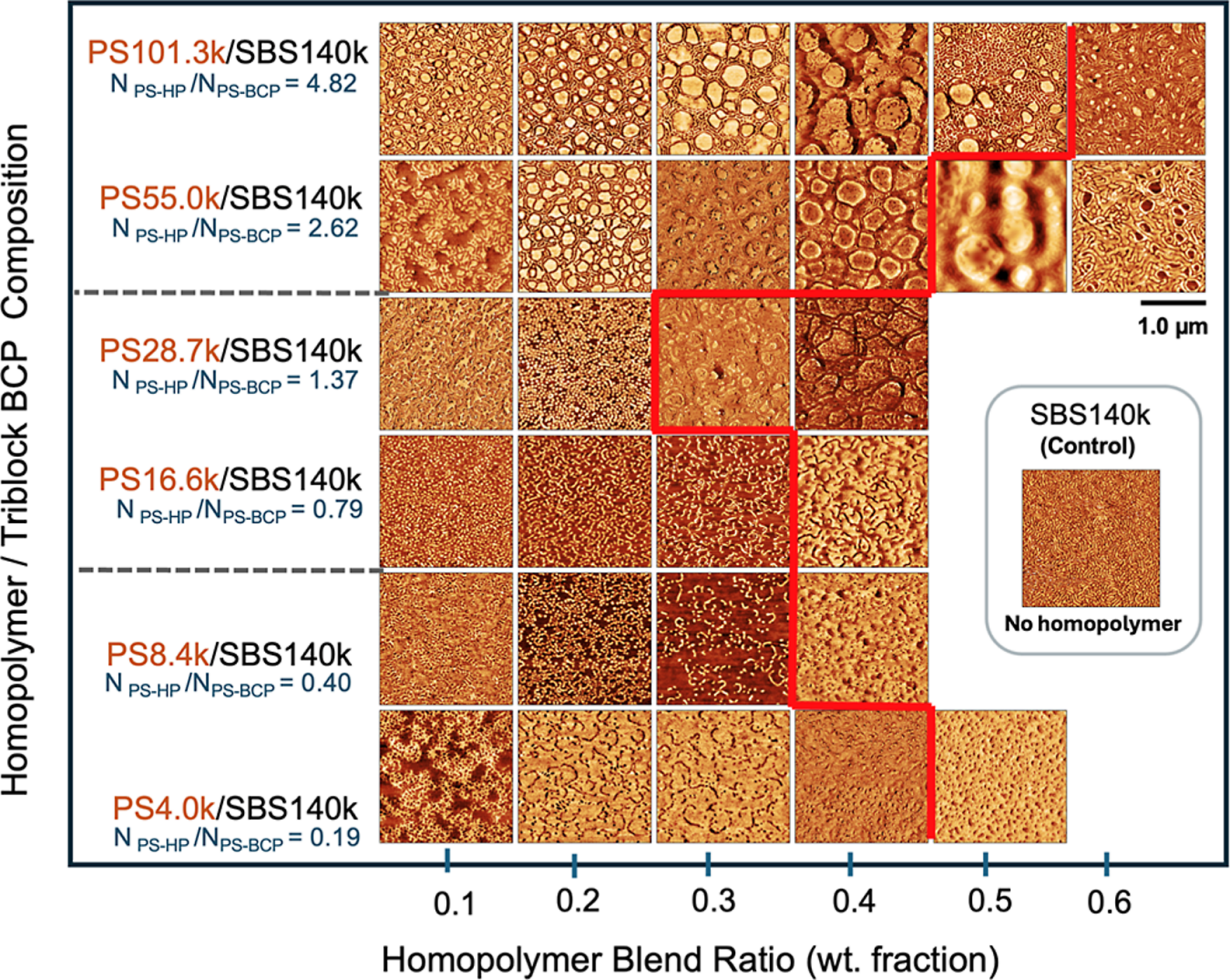
AFM phase images of selected samples of triblock BCP/HP blends. The red line represents order–disorder boundary based on GISAXS data. Image scale (2 μm × 2 μm).

**Figure 6:**
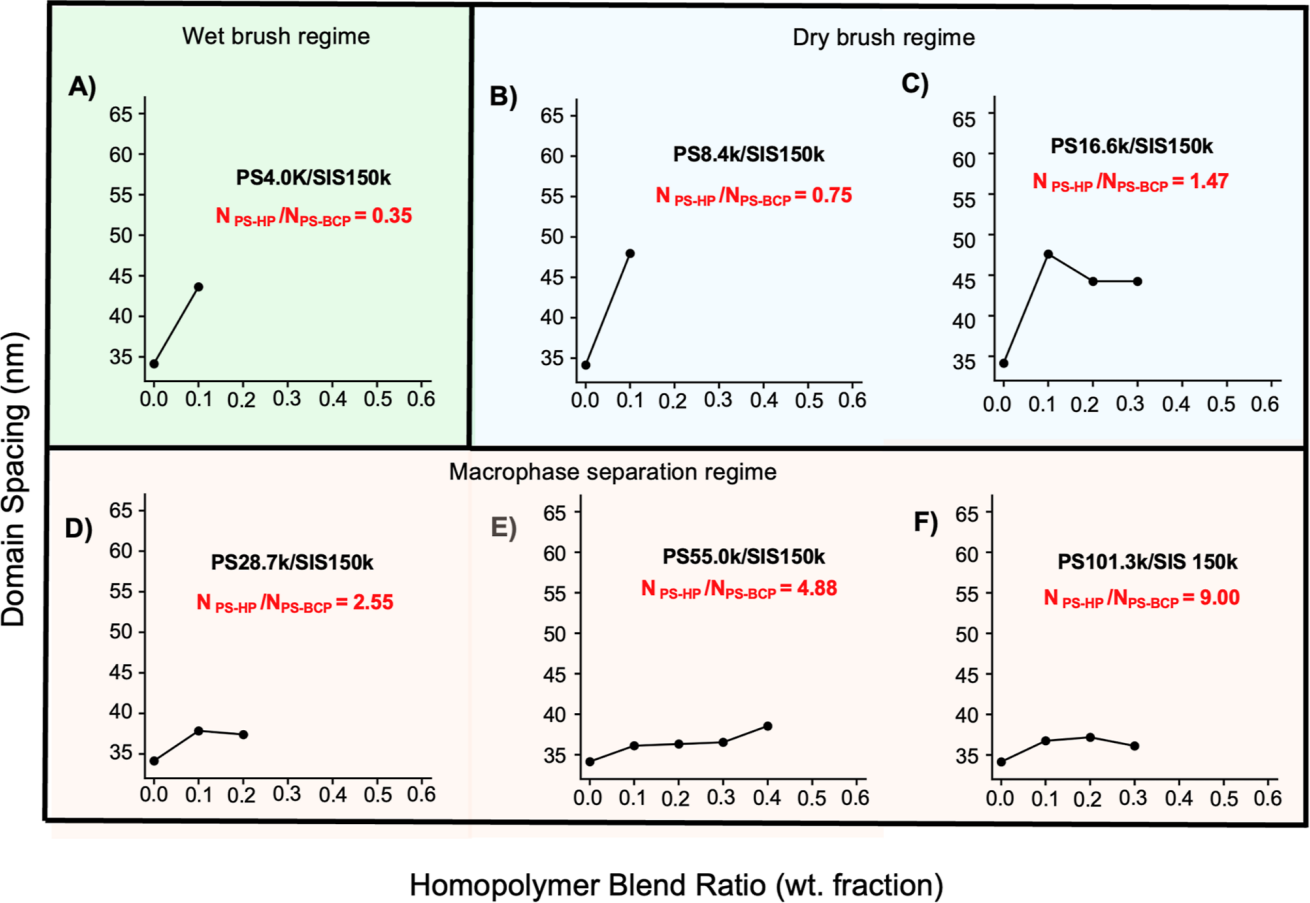
Evolution of domain spacing in PS-*b*-PI-*b*-PS/PS blends as a function of homopolymer molecular weight (MW) and blend ratio (BR). The data reveal three distinct regimes: (A) low-MW homopolymers (wet-brush regime) cause gradual domain swelling, (B,C) medium-MW homopolymers (dry-brush regime) produce pronounced and rapid domain expansion, and (D–F) high-MW homopolymers (macrophase-separation regime) lead to macrophase separation with only minimal changes in spacing.

To complement the inverse/reciprocal space results obtained from X-ray scattering, real-space AFM images were acquired for the BCP/HP blend samples. Figure 5 presents the AFM phase images for SBS/PS blends with varying BRs and PS MWs. While our multiangle GISAXS analysis (Figure S23) confirms robust ordering uniformly throughout the film bulk, the AFM phase images occasionally exhibit surface texturing. This is a common artifact of the free-surface boundary condition in spin-coated films, where preferential segregation of the lower surface energy block to the air interface can form a thin overlayer that partially obscures the underlying bulk cylindrical morphology. The red line in the image array represents the order–disorder boundary identified from the GISAXS data. At low blend ratios with low-MW PS HPs (4.0 and 8.4 kDa), the films exhibited well-ordered porous domains whose spacing increased progressively with HP loading. Medium-MW PS HPs (16.6 and 28.7 kDa) promoted significant domain swelling, leading to isolated cylindrical features and, at higher BR, a complete loss of long-range order consistent with the ODT determined by GISAXS. In contrast, high-MW PS HPs produced macrophase-separated morphologies characterized by irregular featureless domains, confirming their limited compatibility with the triblock BCP.

The CG-MD simulations captured a clear MW-dependent transition in PS/SBS blends. In Figure S22A, low-MW PS homopolymer (8.4 kDa) distributed relatively uniformly throughout the PS domains, whereas medium-MW homopolymer (28.7 kDa) induced stronger domain swelling and preferential enrichment toward the domain interior. Consistently, the trend of domain spacing in Figure S22B increasing nonlinearly with the blend ratio mirrored the wet-brush- and dry-brush-specific experimentally observed swelling trends. The folded composition profiles in Figure S22C further supported the observation in Figure S22A by understanding the relative composition of HP added.

## Conclusion

Using our high-throughput robotic workflow combined with GISAXS and AFM characterization, we systematically mapped the order–disorder transitions in PS-*b*-PB-*b*-PS and PS-*b*-PI-*b*-PS triblock copolymer and PS HPs blends across a broad range of molecular weights. As illustrated in Figure 7, the MW of the HPs with respect to its corresponding domain of triblock BCPs critically dictates both the domain spacing evolution and the onset of disorder. Low-MW HPs (wet-brush) show only a gradual increase in domain spacing before disordering at high blend ratios, whereas medium-MW HPs (dry-brush) exhibit a swift spacing increase followed by earlier disordering. In contrast, high-MW HPs display macrophase separation with negligible changes in spacing, reflecting poor compatibility with the triblock BCP domains. Classical SCFT for diblock/homopolymer blends predicts robust swelling, as the chains are anchored at a single junction. In contrast, the tethered network of bridged and looped conformations in ABA triblocks imposes a severe elastic penalty during domain swelling, driving the rapid loss of long-range order observed here. Furthermore, our observation of kinetically trapped, defective states in the high-MW regime is consistent with theoretical model predictions by Huang et al. and Xie et al. as discussed in the introduction.^39,40^ These models predicted that ABA/A blends are distinctly more susceptible to irregular morphologies and macrophase separation than corresponding diblock systems due to severe topological and packing frustrations. Together, these findings provide clear MW-dependent guidelines for tailoring nanoscale morphologies in multiblock copolymer systems and highlight the power of high-throughput approaches for accelerating soft materials design. This also provides guidance for future material design experiments integrating the grid search knowledge with AI-assisted closed-loop optimization approaches. By informing model predictions with the observed trends, the search space will become more targeted thereby reducing experimental overhead and improving convergence toward optimal morphologies.

**Figure 7:**
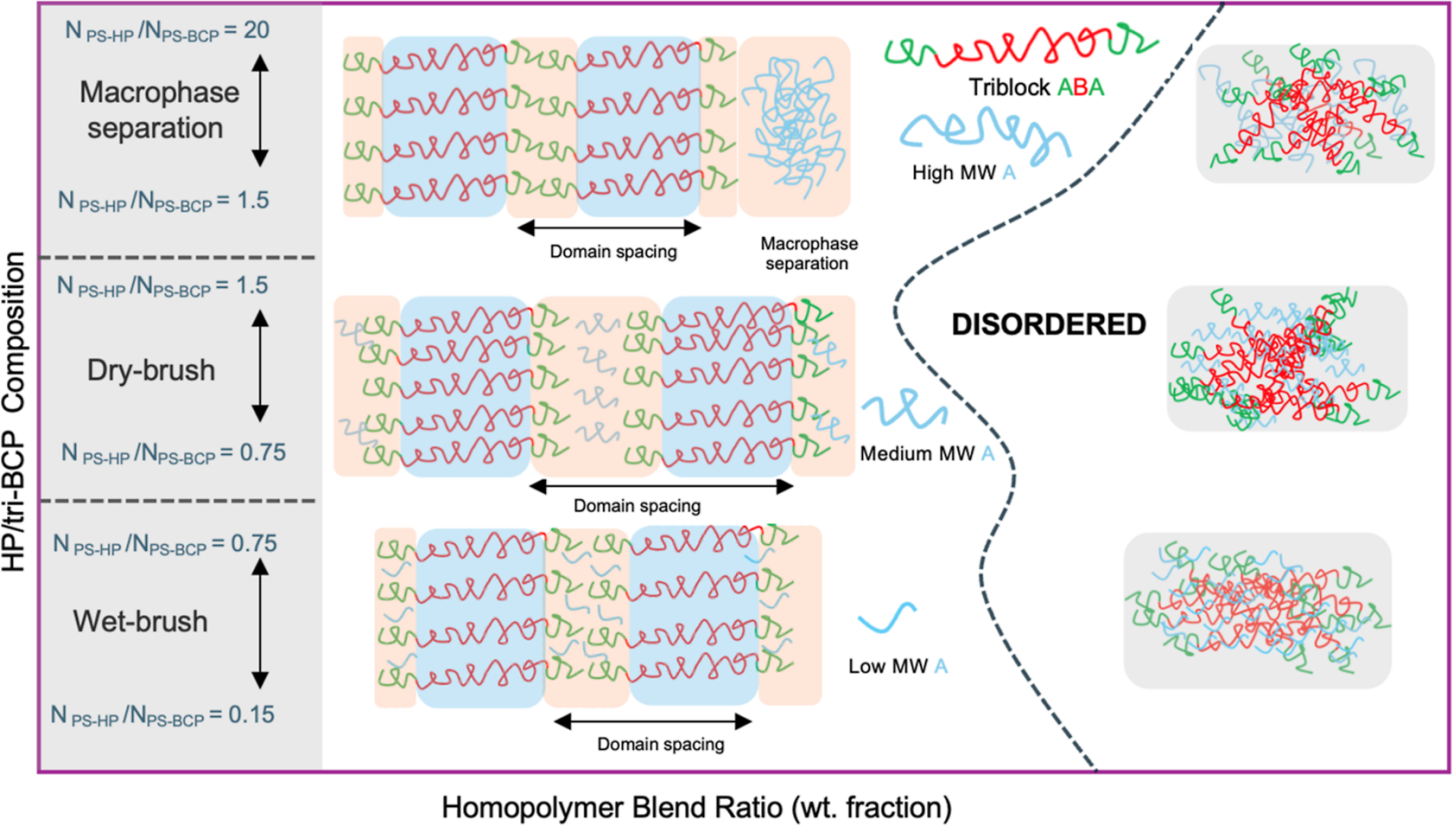
Three different categories of molecular weights of homopolymers identified based on the experimental results: low MW, medium MW, and high MW.

## Experimental Section

### Materials

Polystyrene-block-polybutadiene-block-polystyrene (PS-*b*-PB-*b*-PS) (140.0 kDa, 30% styrene) and polystyrene-block-polyisoprene-block-polystyrene (PS-*b*-PI-*b*-PS) (150.0 kDa, 15% styrene) triblock BCPs were purchased from Scipoly and used as received. All solvents, toluene, tetrahydrofuran, and high-boiling-point solvent additives 1-chloronaphthalene, were purchased from Sigma-Aldrich and used as received.

### Thin Film Sample Preparation

PS-*b*-PB-*b*-PS and PS-*b*-PI-*b*-PS were dissolved in mixture solvents toluene (80% volume) and tetrahydrofuran (THF) (20% volume) to obtain a 2 wt % BCP stock solution. Exactly similar solvent composition and concentration were used to obtain stock solutions of six different MWs of PS HPs (4.0, 8.4, 16.6, 28.7, 55.0, and 101.3 kDa). 1-Chloronaphthalene (boiling point of 259 °C), used as an annealing additive, was diluted to a 16.6% volume fraction in a Tol/THF 80/20% mixture. Silicon wafers with native silicon oxides (Waferpro Inc.) were cleaned for 5 min using oxygen plasma (Diener Inc. at 10 mTorr, 20 sccm O_2_, 40 W). The films were coated using the spin coater of the NOVA sample processing robot at 2000 rpm for 30 s. NOVA is a custom-made robotic platform originally purchased from North Robotics company. The robotic sample formulation workflow has been discussed in the main text of the manuscript.

### Morphology Characterization

AFM images of cast films were collected using a Bruker Dimension Icon 3000 scanning probe microscope in tapping mode. The AFM used a standard Veeco RTESP silicon probe (cantilever length, 125 μm; nominal force constant, 40 N/m; resonance frequency, 350 kHz). Height and phase images were collected simultaneously at a scan rate of 1 Hz with 256 points in each dimension. Images were analyzed using Gwyddion software.

GISAXS measurements were conducted at Complex Material Scattering (CMS) at National Synchrotron Light Source-II (NSLS-II) with an X-ray energy of 13.5 keV. Each sample was exposed to X-ray for 10 s, and 5 different incidence angles were measured from 0.08 to 0.16. The measurement was done under vacuum to reduce air scattering. The scattering results were collected by a Pilatus 1 M detector. The sample to detector distance (∼5 m) was calibrated using Silver Behenate (AgBh) standard, and SciAnalysis (python-based software) was used to reduce 2D to 1D data.

### Overview of the Coarse-Grained Modeling

Experimental PS-*b*-PB-*b*-PS/PS blends were represented as a generic coarse-grained (CG) melt composed of ABA triblock copolymers mixed with A-type homopolymers (HPs). The triblock architecture and composition are held fixed throughout; only the HP is varied in chain length and loading to mirror the experimental design. Experimental variables are mapped to CG inputs under the equal-density assumption: the molecular-weight ratio is represented by the chain-length ratio R = *M*_w,HP_/*M*_*w*,A block_ ≈ *N*_HP_/*N*_*A*_ (with *N*_A_ the length of one A end block), and the blend ratio by weight was approximated as the HP volume fraction ϕ_HP_ ≈BR. The number of triblock chains *n*_BCP_ is fixed (200 ABA chains), and the number of HP chains is adjusted to realize the target ϕ_HP_; the total bead count therefore varies with ϕ_HP_. Given *n*_BCP_ ABA chains of length *N* = 2*N*_A_ + *N*_B_ and an A-type HP length *N*_HP_ = *RN*_A_, the HP chain count required to realize a target ϕ_HP_ is

1 $$n_{HP}=\frac{\phi_{HP}}{1-\phi_{HP}}\frac{N}{N_{HP}}n_{BCP}$$

which enforces $\phi_{HP}=\frac{n_{HP}N_{HP}}{n_{HP}N_{HP}+n_{BCP}N}$ . The simulation HP molecular weights and the corresponding *R* values are summarized in the SI, reports the computed *n*_HP_ for ϕ_HP_ = 0.1–0.9 at the chosen *n*_BCP_,*N*_*A*_, and *N*_*B*_.

Nonbonded interactions follow a truncated-and-shifted Lennard–Jones (LJ) potential between bead types *i*,*j* ∈ {*A*,*B*,HP}

2 $$U_{LJ}\left(r\right)=\{\begin{array}{ll} 4\varepsilon_{ij}\left[{\left(\frac{\sigma_{ij}}{r}\right)}^{12}-{\left(\frac{\sigma_{ij}}{r}\right)}^{6}-{\left(\frac{\sigma_{ij}}{r_{c,ij}}\right)}^{12}+{\left(\frac{\sigma_{ij}}{r_{c,ij}}\right)}^{6}\right], & r<r_{c,ij},\\ 0, & r\geq r_{c,ij}, \end{array}$$

with σ_*ij*_ = σ is the bead diameter, ε_*ij*_ is the well depth, and (unless stated) *r*_*c*,*ij*_ = 2.5σ. Non-bonded interaction strengths were set to ε_AA_ = ε_BB_ = ε_HP,HP_ = 1.0, ε_AB_ = ε_*B*,HP_ = 0.8, and ε_A,HP_ = 1.0, which increases A/B incompatibility while keeping the HP miscible with A. Adjacent beads are connected by a stiff harmonic bond without bond breaking,

3 $$U_{bond}=\frac{1}{2}k_{b}{\left(r-r_{0}\right)}^{2}$$

with *k*_*b*_ = 2500ε/σ^2^ and *r*_0_ = 0.99σ. LJ reduced units are used throughout (σ = 1, ε = 1, *m* = 1); the natural time unit was $\tau =\sigma \sqrt{m/\varepsilon }$ . Equations of motion are integrated with elocity-Verlet at Δ*t* = 0.005τ. Periodic boundary conditions are applied in all directions and electrostatics are absent. Initial configurations were energy-minimized and then assigned velocities at *T*_init_ = 2.0ε/*k*_B_. A multistage iso-*NPT* Nosé–Hoover protocol (i.e., target reduced pressures cycled between *p* = 0 and *p* = 10) was employed to equilibrate the system density and promote microphase separation without introducing biasing lateral stresses. The final production window was performed at *T* = 0.7 and *p* = 0 for 10^6^ steps following several shorter equilibration stages; thermostat and barostat damping times are 1.0τ and 5.0τ, respectively. Thermodynamic data are sampled every 10^4^ steps and coordinates saved periodically for analysis.

All simulations were initialized under periodic boundary conditions in a cubic box with bounds *x*,*y*,*z* ∈ [−21.077, 21.077], corresponding to *L*_*x*_ = *L*_*y*_ = *L*_*z*_ = 42.154 in reduced LJ units. Equilibration was assessed by monitoring both the mass density and the total potential energy, and production analyses were carried out only after these quantities reached steady plateaus with no systematic drift. To make potential finite-size concerns for Figure S22, each snapshot shows the entire simulation cell, and all reported structural metrics (domain spacing and folded composition profiles) were computed from the equilibrated trajectories rather than inferred from visual inspection alone.

The local PS fraction from simulation (Figure S22) was defined as

4 $$\phi_{PS}\left(s\right)=\frac{{\mathrm{\rho ̃}}_{HP}\left(s\right)+{\mathrm{\rho ̃}}_{PS,BCP}\left(s\right)}{{\mathrm{\rho ̃}}_{HP}\left(s\right)+{\mathrm{\rho ̃}}_{PS,BCP}\left(s\right)+{\mathrm{\rho ̃}}_{B}\left(s\right)}$$

PS-rich regions were identified by ϕ_PS_(*s*) ≥ ϕ_th_. To quantify the spatial distribution of HP within the PS domains independent of domain width, we computed

5 $$f_{HP}\left(s\right)=\frac{{\mathrm{\rho ̃}}_{HP}\left(s\right)}{{\mathrm{\rho ̃}}_{HP}\left(s\right)+{\mathrm{\rho ̃}}_{PS,BCP}\left(s\right)+\epsilon^{\prime }}$$

where *ϵ*′ is a small constant to avoid division by zero. A nearly constant *f*_HP_(*s*) within PS-rich regions indicates a uniform HP distribution, whereas a pronounced peak toward the domain interior indicates core-localized enrichment.

## Supplementary Material





## Notes

Supplementary figures of the NOVA robotic platform, thermal vs NVASA annealing phase maps, full 2D GISAXS scattering image arrays, native morphological characterization, domain spacing comparisons, CG-MD simulation profiles, and summary tables of theoretical parameters and structural data (PDF)
Demonstration video illustrating the robotic high-throughput workflow in detail (MP4)

## References

[1] Cummins C., Lundy R., Walsh J., Ponsinet V., Fleury G., Morris M. (2020). Enabling Future Nanomanufacturing through Block Copolymer
Self-Assembly: A review. Nano Today.

[2] Zhou H., Yang G.-W., Zhang Y.-Y., Xu Z. K., Wu G. (2018). Bioinspired
Block Copolymer for Mineralized Nanoporous Membrane. ACS Nano.

[3] Feng H., Dolejsi M., Zhu N., Yim S., Loo W., Ma P., Zhou C., Craig G. S. W., Chen W., Wan L., Ruiz R., de Pablo J. J., Rowan S. J. (2022). Optimized
Design of Block Copolymers with Covarying Properties for Nanolithography. Nat. Mater..

[4] Xiong S., Wan L., Ishida Y., Chapuis Y., Craig G., Ruiz R., Nealey P. (2016). Directed Self-Assembly
of Triblock Copolymer on Chemical
Patterns for Sub-10-nm Nanofabrication via Solvent Annealing. ACS Nano.

[5] Song D., Li C., Li W., Watkins J. (2016). Block Copolymer Nanocomposites with
High Refractive Index Contrast for One-Step Photonics. ACS Nano.

[6] Yang Y., Kim H., Xu J., Hwang M.-S., Tian D., Wang K., Zhang L., Liao Y., Park H.-G., Yi G. (2018). Responsive Block Copolymer Photonic Microspheres. Adv. Mater..

[7] Dutta D., Ke W., Xi L., Yin W., Zhou M., Ge Z. (2019). Block Copolymer
Prodrugs: Synthesis, Self-Assembly, and Applications for Cancer Therapy. Wiley Interdiscip. Rev.: Nanomed. and Nanobiotechnol..

[8] Patel D., Kuperkar K., Yusa S., Bahadur P. (2023). Nanoscale Self-Assemblies
from Amphiphilic Block Copolymers as Proficient Templates in Drug
Delivery. Drugs Drug Candidates.

[9] Leibler L. (1980). Theory of
Microphase Separation in Block Copolymers. Macromolecules.

[10] Matsen M. (2020). Field Theoretic
Approach for Block Polymer Melts: SCFT and FTS. J. Chem. Phys..

[11] Vigil D., Quah T., Sun D., Delaney K., Fredrickson G. (2022). Self-Consistent
Field Theory Predicts Universal Phase Behavior for Linear, Comb, and
Bottlebrush Diblock Copolymers. Macromolecules.

[12] Zhang J., Clark M., Wu C., Li M., Trefonas P., Hustad P. (2016). Orientation Control in Thin Films
of a High-χ
Block Copolymer with a Surface Active Embedded Neutral Layer. Nano Lett..

[13] Oh J., Suh H., Ko Y., Nah Y., Lee J. C., Yeom B., Char K., Ross C., Son J. (2019). Universal Perpendicular
Orientation of Block Copolymer Microdomains Using a Filtered Plasma. Nat. Commun..

[14] Gu X., Gunkel I., Hexemer A., Gu W., Russell T. P. (2014). An In Situ
Grazing Incidence X-Ray Scattering Study of Block Copolymer Thin Films
During Solvent Vapor Annealing. Adv. Mater..

[15] Doerk G. S., Yager K. G. (2023). Diversifying Self-Assembled Phases
in Block Copolymer
Thin Films via Blending. Phys. Rev. Mater..

[16] Zhang H., Clothier G. K., Guimarães T. R., Kita R., Zetterlund P. B., Okamura Y. (2022). Tuning Phase Separation
Morphology in Blend Thin Films
Using Well-Defined Linear (multi) Block Copolymers. Polymer.

[17] Chen H., Chakrabarti A. (1998). Morphology
Of Thin Block Copolymer Films on Chemically
Patterned Substrates. J. Chem. Phys..

[18] Lin Y.-H., Shiu C.-C., Chen T.-L., Chen H.-L., Tsai J.-C. (2021). Solubilization
Behavior of Homopolymer in Its Blend with the Block Copolymer Displaying
the Feature of Lower Critical Ordering Transition. Polymers.

[19] Matsen M. W. (1995). Phase Behavior
of Block Copolymer/Homopolymer Blends. Macromolecules.

[20] Whitmore M., Vavasour J. (1995). Self-Consistent Field Theory of Block
Copolymers and
Block Copolymer Blends. Acta Polym..

[21] Owens J., Gancarz I., Koberstein J., Russell T. (1989). Order-Disorder Transitions
in Mixtures of Homopolymers with Diblock Copolymers. Macromolecules.

[22] Goodson A. D., Liu G., Rick M. S., Raymond A. W., Uddin M. F., Ashbaugh H. S., Albert J. N. (2019). Nanostructure Stability and Swelling of Ternary Block
Copolymer/Homopolymer Blends: A Direct Comparison between Dissipative
Particle Dynamics and Experiment. J. Polym.
Sci., Part B: Polym. Phys..

[23] Kim Y., Mun J., Yu G., Char K. (2017). Phase Transition of Block Copolymer/Homopolymer
Binary Blends under 2D Confinement. Macromol.
Res..

[24] Rokhlenko Y., Moschovas D., Miskaki C., Chan E. P., Avgeropoulos A., Osuji C. O. (2019). Creating Aligned Nanopores by Magnetic Field Processing
of Block Copolymer/Homopolymer Blends. ACS Macro
Lett..

[25] Greenall M. J., Buzza D. M. A., McLeish T. C. (2009). Micelle
Formation in Block Copolymer/Homopolymer
Blends: Comparison of Self-Consistent Field Theory with Experiment
and Scaling Theory. Macromolecules.

[26] Ly D. Q., Makatsoris C. (2019). Effects of the Homopolymer Molecular Weight on a Diblock
Copolymer in a 3D Spherical Confinement. BMC
Chem..

[27] Martínez-Veracoechea F. J., Escobedo F. A. (2007). Monte Carlo Study
of the Stabilization of Complex Bicontinuous
Phases in Diblock Copolymer Systems. Macromolecules.

[28] Koizumi S., Hasegawa H., Hashimoto T. (1994). Ordered Structures of Block Copolymer/Homopolymer
Mixtures. 5. Interplay of Macro and Microphase Transitions. Macromolecules.

[29] Orso K. A., Green P. F. (1999). Phase Behavior of Thin Film Blends of Block Copolymers
and Homopolymers: Changes in Domain Dimensions. Macromolecules.

[30] Choi C., Ahn S., Kim J. K. (2020). Diverse
Morphologies of Block Copolymers by Blending
with Homo (and Co) Polymers. Macromolecules.

[31] Sun Y.-S., Liao Y.-P., Hung H.-H., Chiang P.-H., Su C.-J. (2024). Molecular-Weight
Effects of a Homopolymer on the AB and ABC Stacks of Perforations
in Block Copolymer/Homopolymer Films. Soft Matter.

[32] Doerk G. S., Yager K. G. (2017). Rapid Ordering in ″Wet Brush″ Block Copolymer/Homopolymer
Ternary Blends. ACS Nano.

[33] Spontak R. J., Smith S. D., Ashraf A. (1993). Dependence of the OBDD Morphology
on Diblock Copolymer Molecular Weight in Copolymer/Homopolymer Blends. Macromolecules.

[34] Peng J., Gao X., Wei Y., Wang H., Li B., Han Y. (2005). Controlling
the Size of Nanostructures in Thin Films via Blending of Block Copolymers
and Homopolymers. J. Chem. Phys..

[35] Toth K., Bae S., Osuji C. O., Yager K. G., Doerk G. S. (2021). Film Thickness and
Composition Effects in Symmetric Ternary Block Copolymer/Homopolymer
Blend Films: Domain Spacing and Orientation. Macromolecules.

[36] Qiang X., Chakroun R., Janoszka N., Gröschel A. H. (2019). Self-Assembly
of Multiblock Copolymers. Isr. J. Chem..

[37] Abdelrahman D., Iseli R., Musya M., Jinnai B., Fukami S., Yuasa T., Sai H., Wiesner U. B., Saba M., Wilts B. D. (2023). Directed Self-Assembly
of Diamond Networks
in Triblock Terpolymer Films on Patterned Substrates. ACS Appl. Mater. Interfaces.

[38] Chen K., Wang F., Liu M., Wang X. (2022). Tunable Helical Structures
Formed by Blending ABC Triblock Copolymers and C Homopolymers in Nanopores. Polym. Int..

[39] Xie J., Shi A. (2024). C. Phase Behavior of
Triblock Copolymer and Homopolymer Blends: Effect
of Copolymer Topology. Phys. Rev. Mater..

[40] Huang Y., Xia H., Liu H., Hu Y. (2007). Monte-Carlo Study of Triblock Copolymer/Homopolymer
Blend Films. Macromol. Theory Simul..

[41] Langner S., Häse F., Perea J. D., Stubhan T., Hauch J., Roch L. M., Heumueller T., Aspuru-Guzik A., Brabec C. J. (2020). Beyond Ternary OPV: High-Throughput
Experimentation
and Self-Driving Laboratories Optimize Multicomponent Systems. Adv. Mater..

[42] Bai Y., Wilbraham L., Slater B. J., Zwijnenburg M. A., Sprick R. S., Cooper A. I. (2019). Accelerated
Discovery of Organic
Polymer Photocatalysts for Hydrogen Evolution from Water through the
Integration of Experiment and Theory. J. Am.
Chem. Soc..

[43] Greenaway R., Santolini V., Bennison M., Alston B., Pugh C., Little M., Miklitz M., Eden-Rump E., Clowes R., Shakil A. (2018). High-Throughput Discovery
of Organic Cages and Catenanes Using Computational Screening Fused
with Robotic Synthesis. Nat. Commun..

[44] Meier M. A., Hoogenboom R., Schubert U. S. (2004). Combinatorial Methods,
Automated
Synthesis and High-Throughput Screening in Polymer Research: The Evolution
Continues. Macromol. Rapid Commun..

[45] Wang C., Kim Y.-J., Vriza A., Batra R., Baskaran A., Shan N., Li N., Darancet P., Ward L., Liu Y. (2025). Autonomous Platform for Solution Processing of Electronic
Polymers. Nat. Commun..

[46] Beaucage P. A., Martin T. B. (2023). The Autonomous Formulation Laboratory: An Open Liquid
Handling Platform for Formulation Discovery Using X-ray and Neutron
Scattering. Chem. Mater..

[47] Quinn H., Robben G. A., Zheng Z., Gardner A. L., Werner J. G., Brown K. A. (2024). PANDA: A Self-Driving Lab for Studying
Electrodeposited
Polymer Films. Mater. Horiz..

[48] MacLeod B. P., Parlane F. G., Morrissey T. D., Häse F., Roch L. M., Dettelbach K. E., Moreira R., Yunker L. P., Rooney M. B., Deeth J. R. (2020). Self-Driving Laboratory
for Accelerated Discovery of Thin-Film Materials. Sci. Adv..

[49] Lamb B., Upreti S., Wang Y., Struble D., Zhu C., Freychet G., Gu X., Ma B. (2026). Machine Learning Framework
for Characterizing Processing-Structure Relationship in Block Copolymer
Thin Films. Macromolecules.

[50] Weller D. W., Galuska L., Wang W., Ehlenburg D., Hong K., Gu X. (2019). Roll-to-Roll Scalable Production
of Ordered Microdomains through Nonvolatile Additive Solvent Annealing
of Block Copolymers. Macromolecules.

[51] Koizumi S., Hasegawa H., Hashimoto T. (1994). Spatial Distribution
of Homopolymers
in Block Copolymer Microdomains as Observed by a Combined SANS and
SAXS Method. Macromolecules.

[52] Sik
Park D., Sancaktar E. (2012). Structural Parameters for Nanocylinder Microdomains
of Polystyrene-Polybutadiene-Polystyrene Triblock Copolymer and Its
Blends with Polystyrene Homopolymer. Curr. Nanosci..

[53] López-Barrón C. R., Eberle A. P., Yakovlev S., Bons A.-J. (2016). Structural Origins
of Mechanical Properties and Hysteresis in SIS Triblock Copolymers/Polystyrene
Blends with Spherical Morphology. Rheol. Acta.

