# Supplementary material for: A Robotic High-Throughput Grid-Search Platform for Mapping Phase Behavior in Triblock Copolymer–Homopolymer Blends
Source: ACS Nano. 2026 Jun 9;20(24):17303–15. doi: 10.1021/acsnano.6c01299 (PMC13296598; doi:10.1021/acsnano.6c01299)
Supplement: Supplementary file 1 [file nn6c01299_si_001.pdf]

Supporting Information

A Robotic High-Throughput Grid-Search Platform for Mapping Phase Behavior in Triblock Copolymer–Homopolymer Blends

Saroj Upreti[1], Lan Xu[2], Md. Moniruzzaman[1], Yunfei Wang[1,3], Kailash Adhikari[1], Sabin Baral[1], Derek Patton[1], Boran Ma[1], Jie Xu[4], Ruipeng Li[5], Chenhui Zhu[3], Wenjie Xia[2], Xiaodan Gu[1*]

1. School of Polymer Science and Engineering, Center for Optoelectronic Materials and Devices, University of Southern Mississippi, Hattiesburg, MS 39406, USA
2. Department of Aerospace Engineering, Iowa State University, Ames, IA 500121, USA
3. Advanced Light Source, Lawrence Berkeley National Laboratory, Berkeley, CA 94720, USA
4. Nanoscience and Technology Division, Argonne National Laboratory, Lemont, IL 60439, USA
5. National Synchrotron Light Source II (NSLS-II), Brookhaven National Lab, Upton, NY 11973, USA

Corresponding email: xiaodan.gu@usm.edu

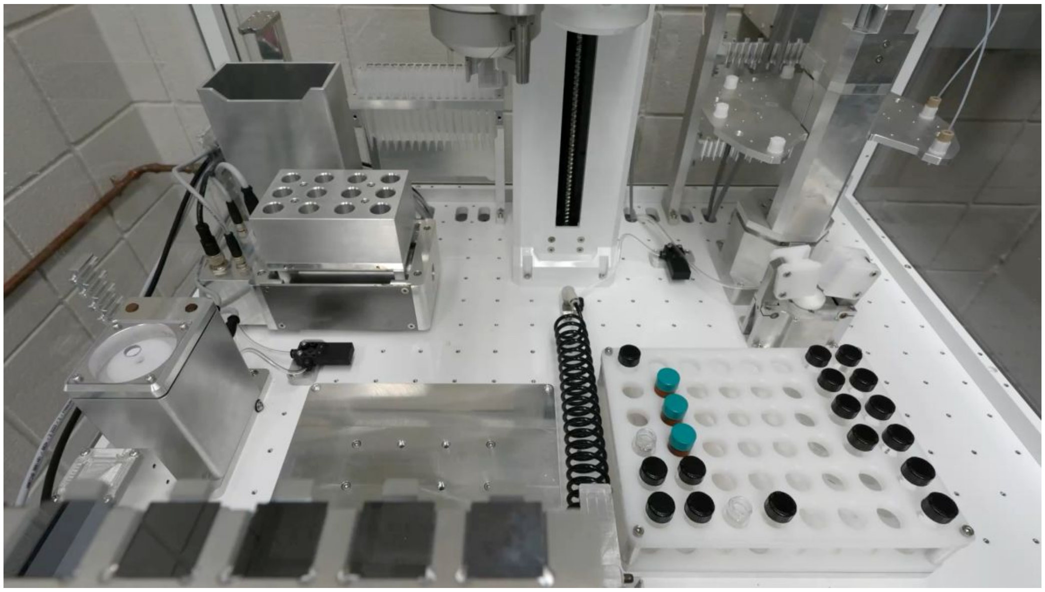

Figure S1: An image of Nova processing robot at the USM campus for high throughput copolymer sample processing.

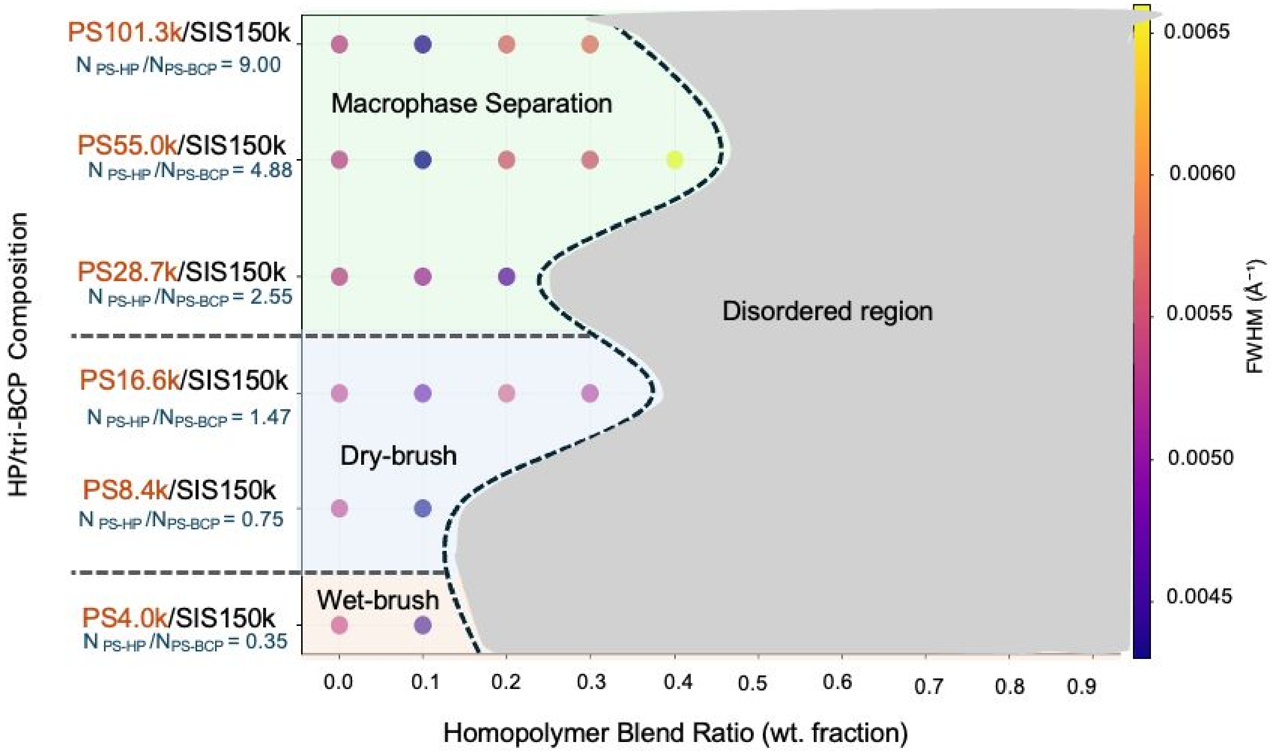

Figure S2: Phase map outlining the ODT boundary for **PS-b-PI-b-PS 150k (SIS)** blended with PS homopolymers at swell ratio of 3. The solid circular markers denote blends exhibiting a distinct primary scattering peak (ordered or macrophase-separated regimes). The color of these solid markers directly correlates to the Full Width at Half Maximum (FWHM) of the primary peak, as indicated by the right-hand color bar. Grey covered area indicates the disordered region that lack a distinct primary scattering peak.

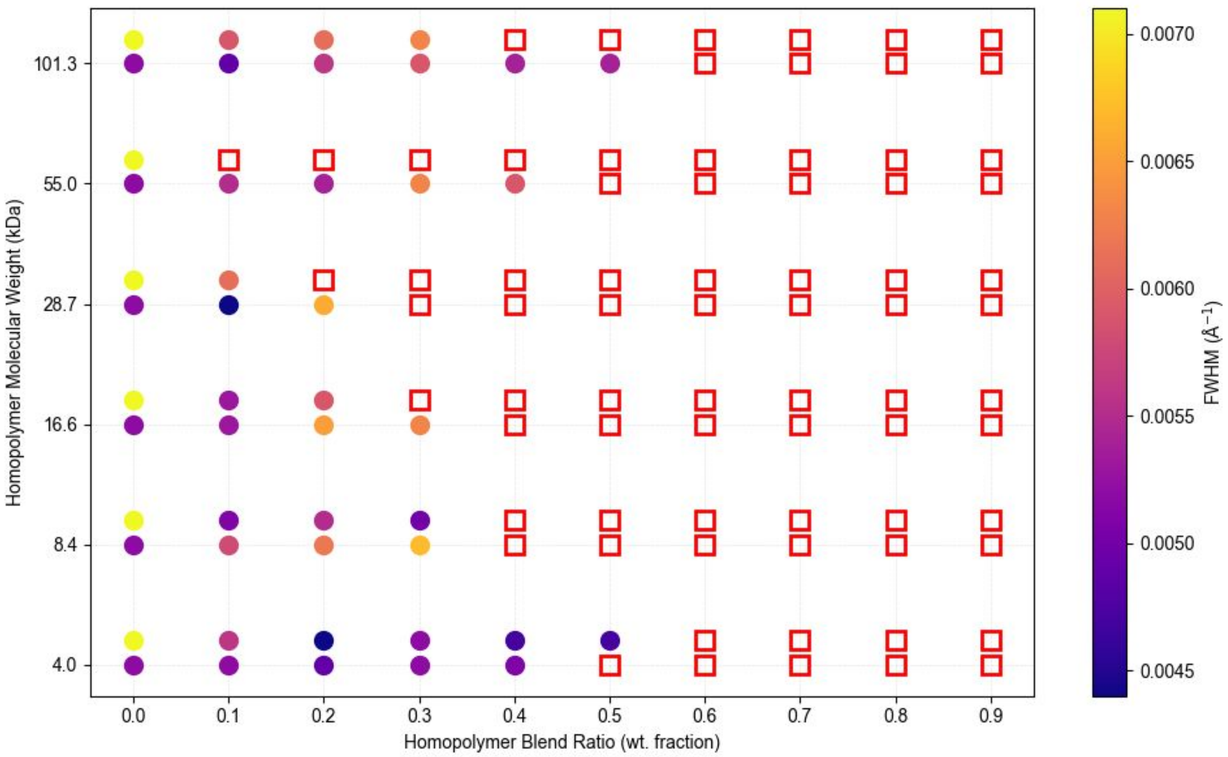

Figure S3: FWHM for thermal annealing (top), NVASA annealing (bottom) at swell ratio of 3 for PS-b-PB-b-PS (**SBS**) triblock copolymers thin film blended with PS homopolymer with different molecular weights. The solid circular markers denote blends exhibiting a distinct primary scattering peak (ordered or macrophase-separated regimes). The color of these solid markers directly correlates to the Full Width at Half Maximum (FWHM) of the primary peak, as indicated by the right-hand color bar. Red squares indicate the disordered region that lack a distinct primary scattering peak.

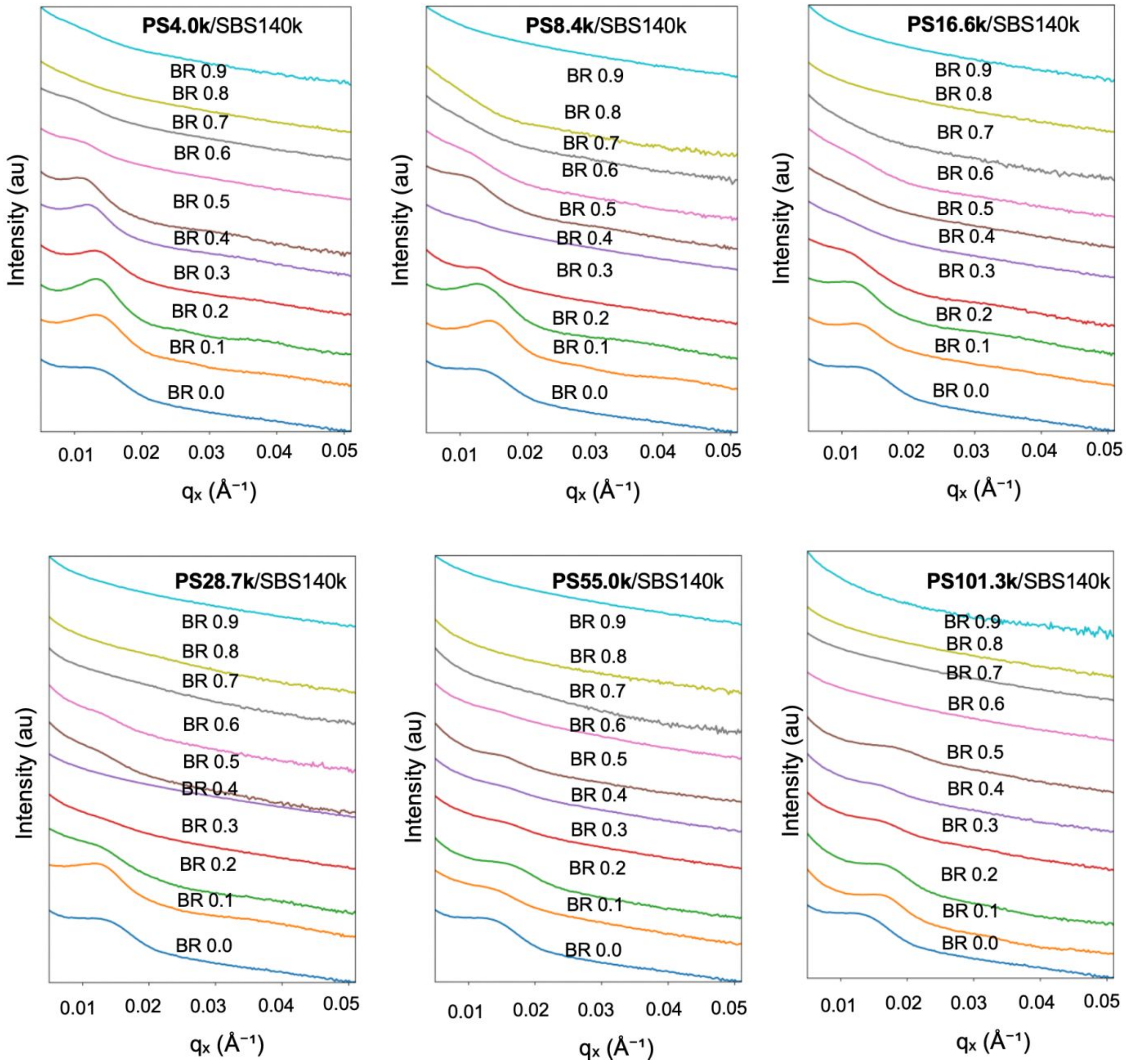

Figure S4: Scattering intensity vs scattering vector curve for thermally annealed PS-b-PB-b-PS (**SBS**) triblock copolymer with PS homopolymers.

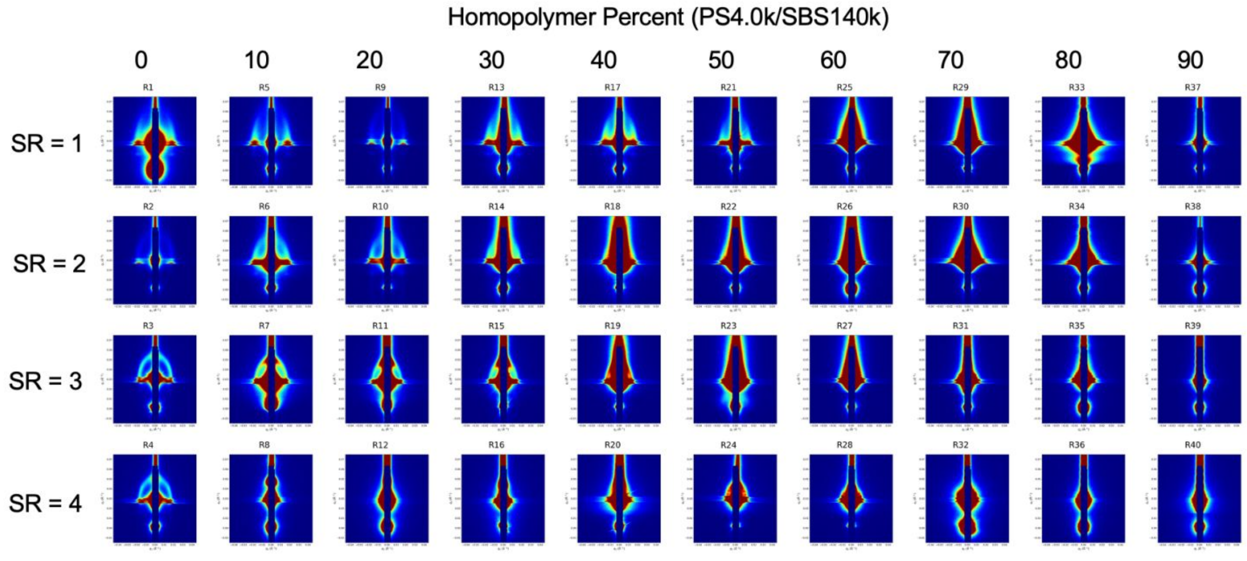

Figure S5: 2D scattering images for PS 4.0k with SBS 140k with different mixing ratios and swell ratios (SR 1~4). Full resolution images are available in an online database.

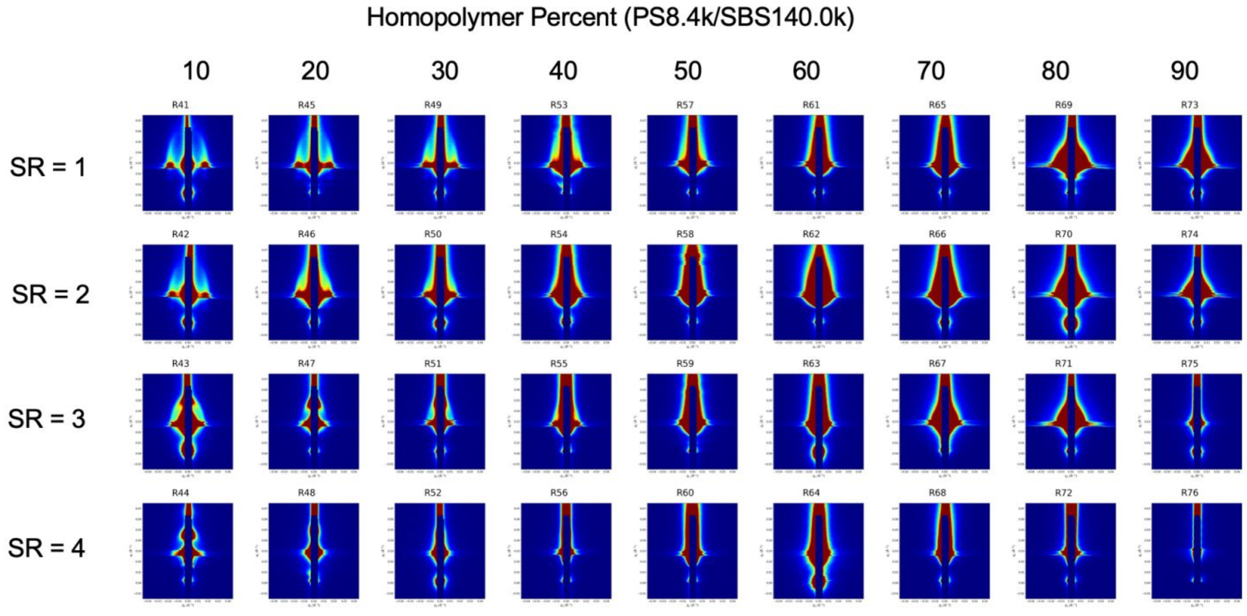

Figure S6: 2D scattering images for PS 8.4k with SBS 140k with different mixing ratios and swell ratios (SR 1~4). Full resolution images are available in an online database.

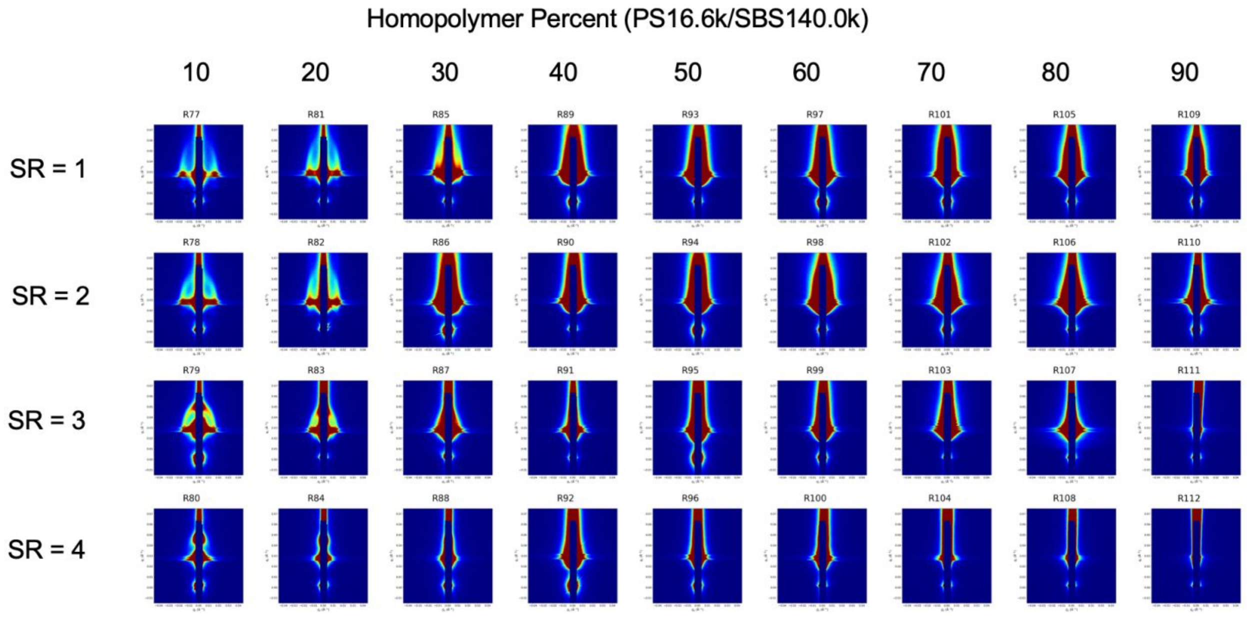

Figure S7: 2D scattering images for PS 16.6k with SBS 140k with different mixing ratios and swell ratios (SR 1~4). Full resolution images are available in an online database.

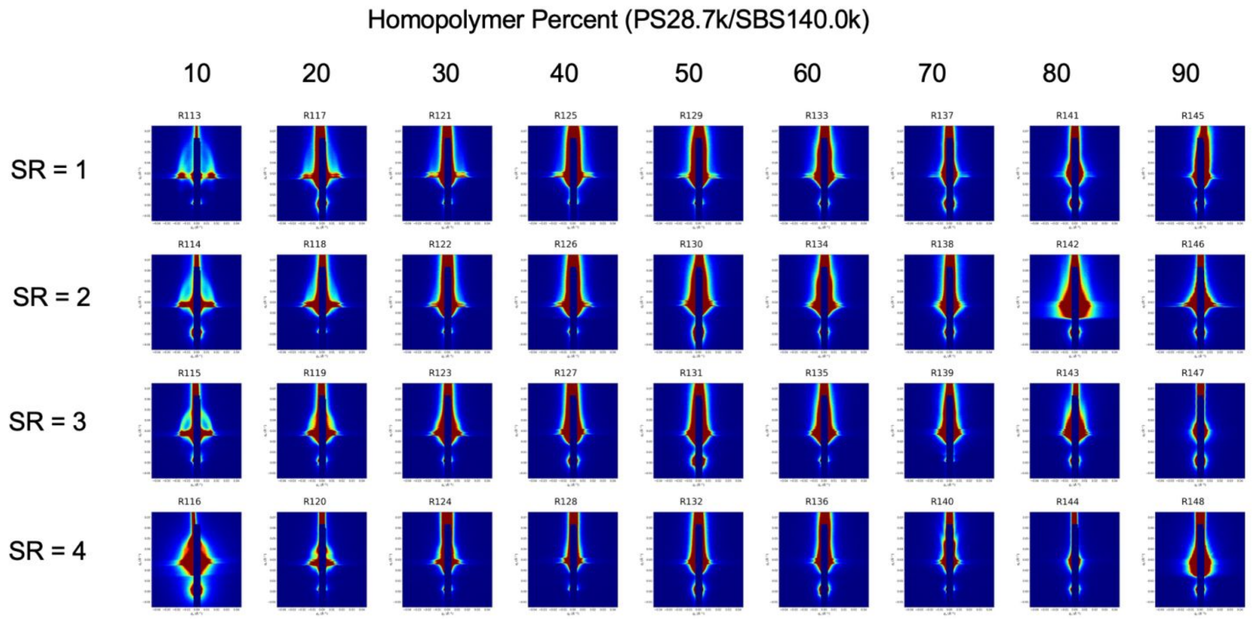

Figure S8: 2D scattering images for PS 28.7k with SBS 140k with different mixing ratios and swell ratios (SR 1~4). Full resolution images are available in an online database.

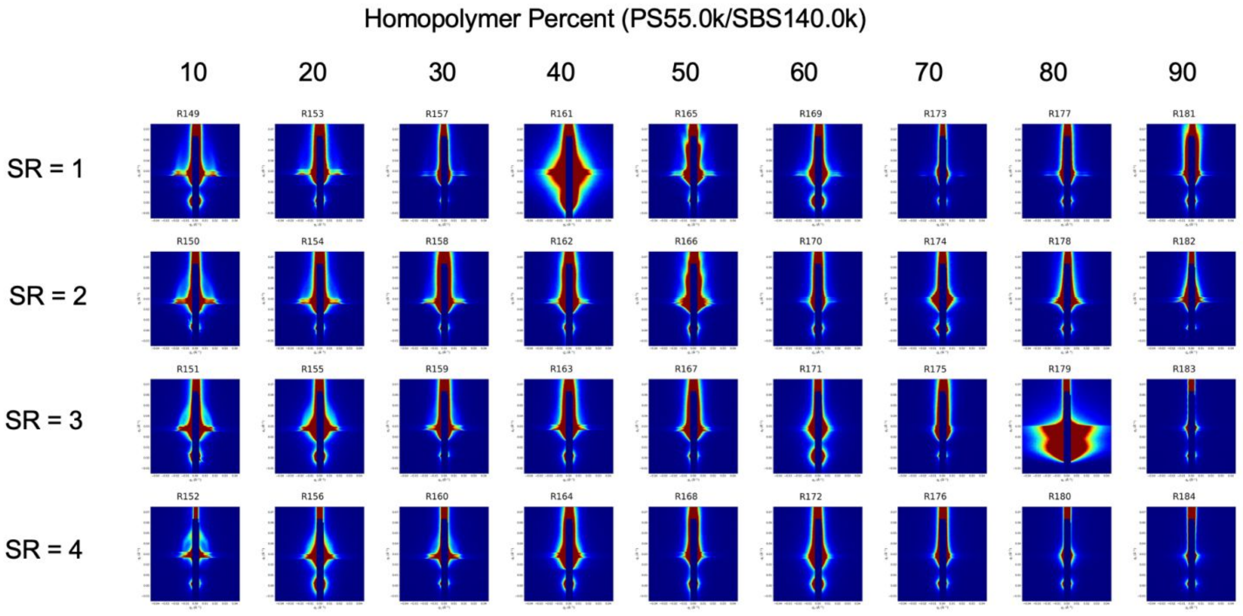

Figure S9: 2D scattering images for PS 55.0k with SBS 140k with different mixing ratios and swell ratios (SR 1~4). Full resolution images are available in an online database.

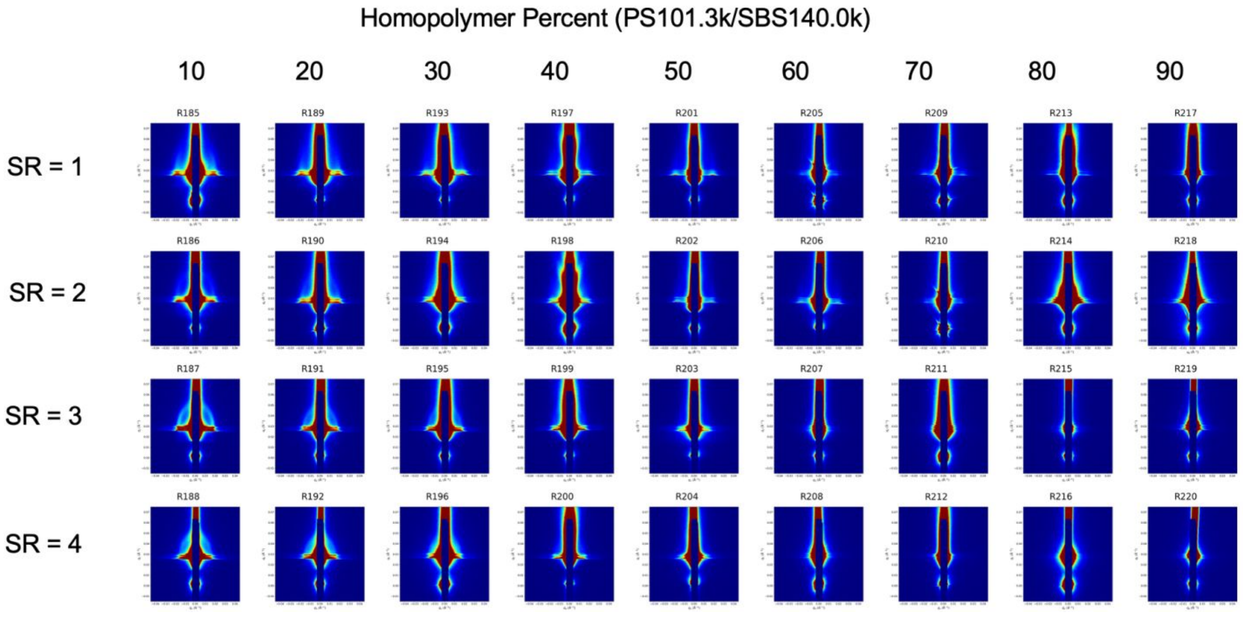

Figure S10: 2D scattering images for PS 101.3k with SBS 140k with different mixing ratios and swell ratios (SR 1~4). Full resolution images are available in an online database.

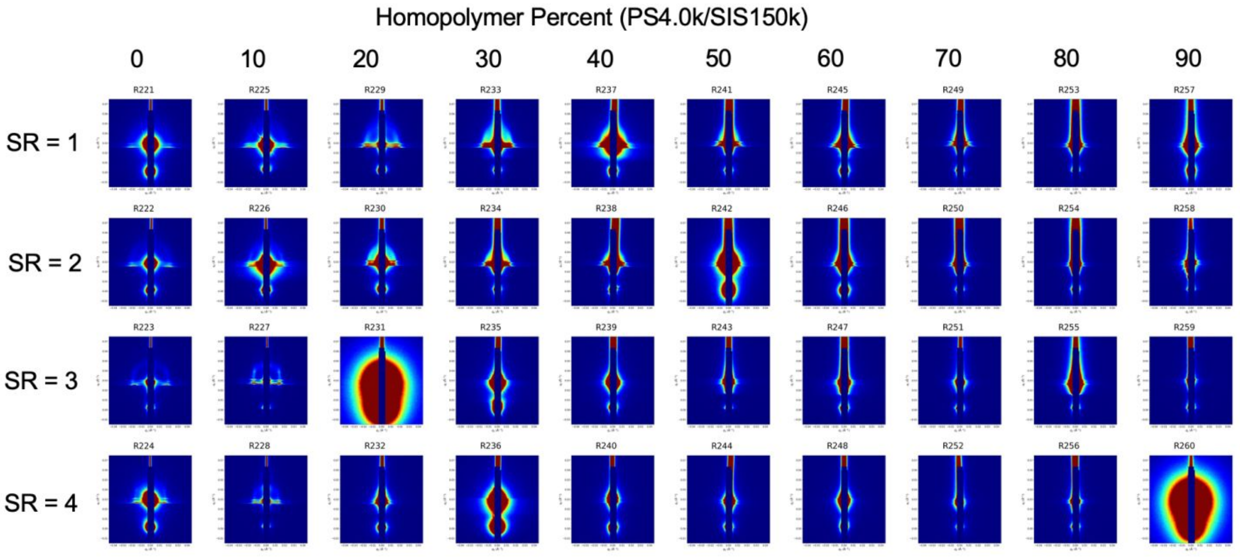

Figure S11: 2D scattering images for **PS 4.0k** with SIS150k with different mixing ratios and swell ratios (SR 1~4). Full resolution images are available in an online database.

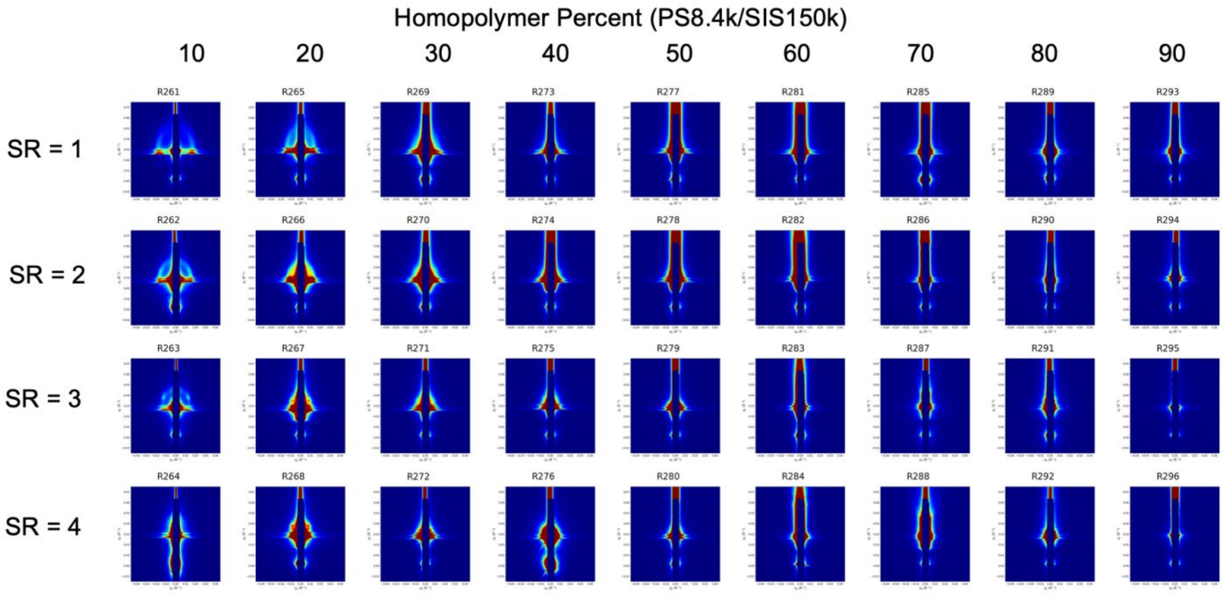

Figure S12: 2D scattering images for **PS 8.4k** with SIS150k with different mixing ratios and swell ratios (SR 1~4). Full resolution images are available in an online database.

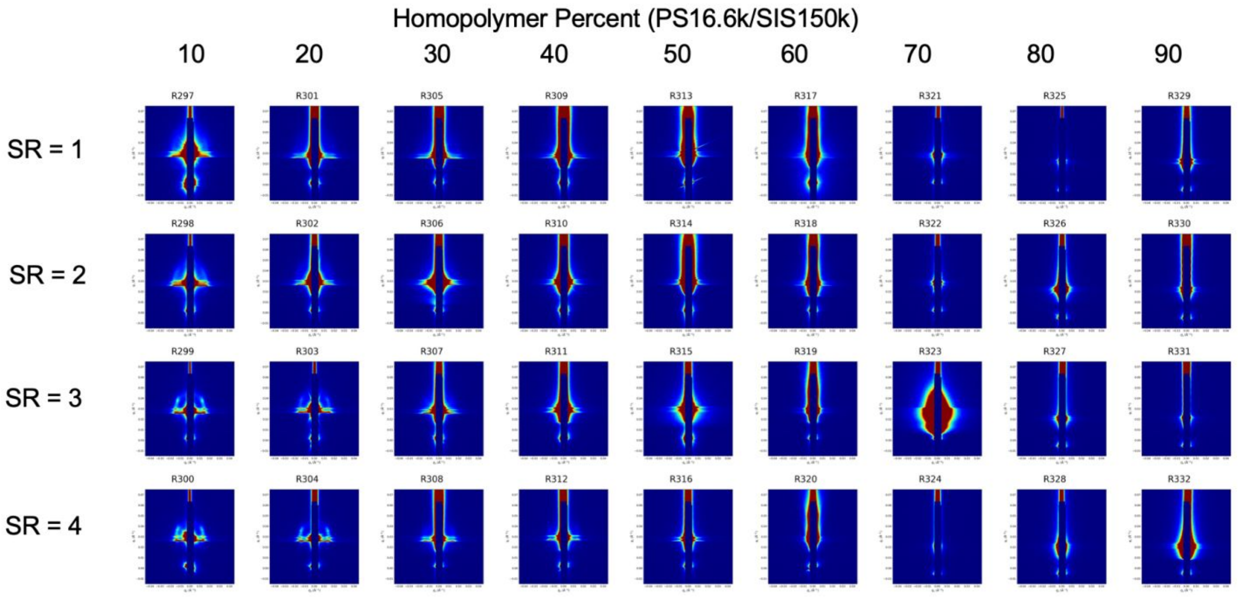

Figure S13: 2D scattering images for **PS 16.6k** with SIS150k with different mixing ratios and swell ratios (SR 1~4). Full resolution images are available in an online database.

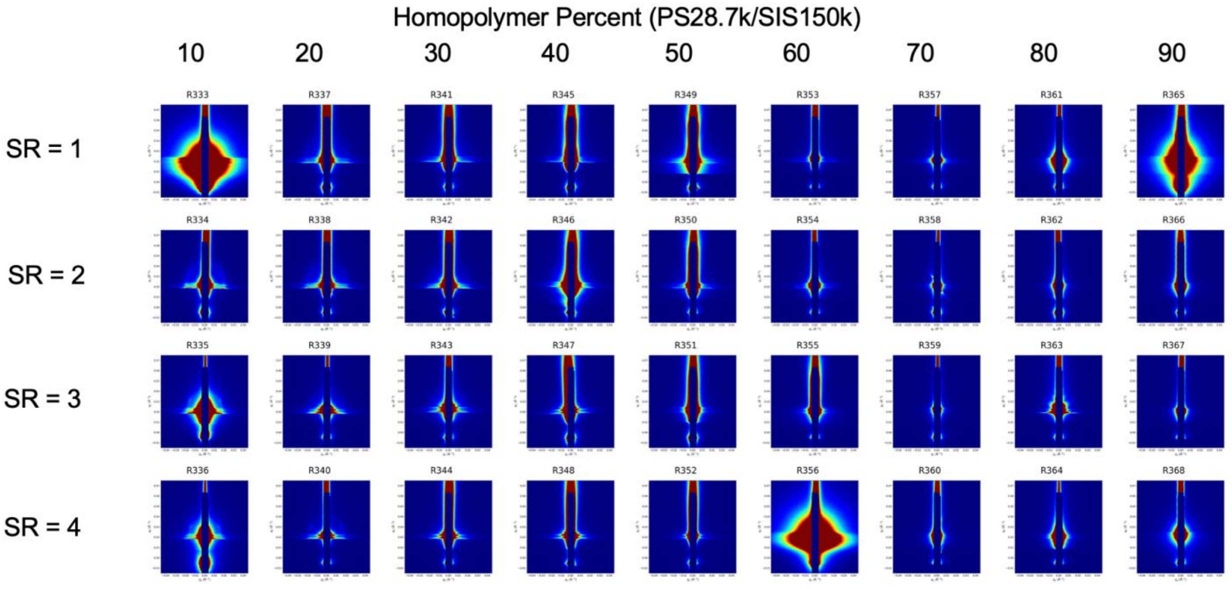

Figure S14: 2D scattering images for **PS 28.7k** with SIS150k with different mixing ratios and swell ratios (SR 1~4). Full resolution images are available in an online database.

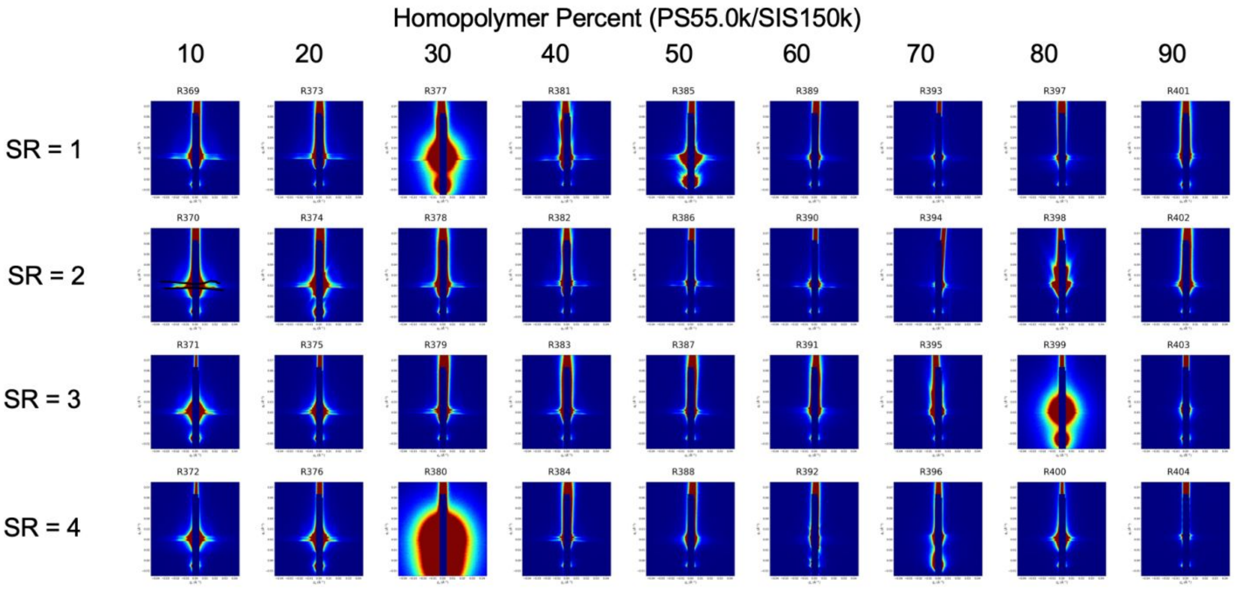

Figure S15: 2D scattering images for **PS 55.0k** with SIS150k with different mixing ratios and swell ratios (SR 1~4). Full resolution images are available in an online database.

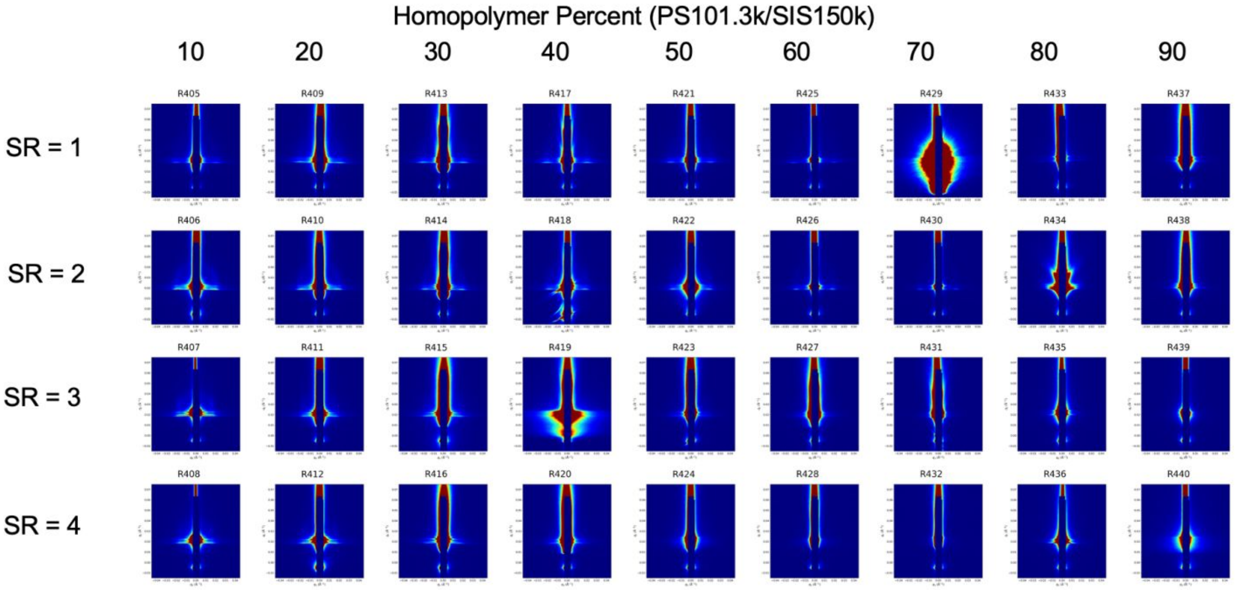

Figure S16: 2D scattering images for **PS 101.3k** with SIS150k with different mixing ratios and swell ratios (SR 1~4). Full resolution images are available in an online database.

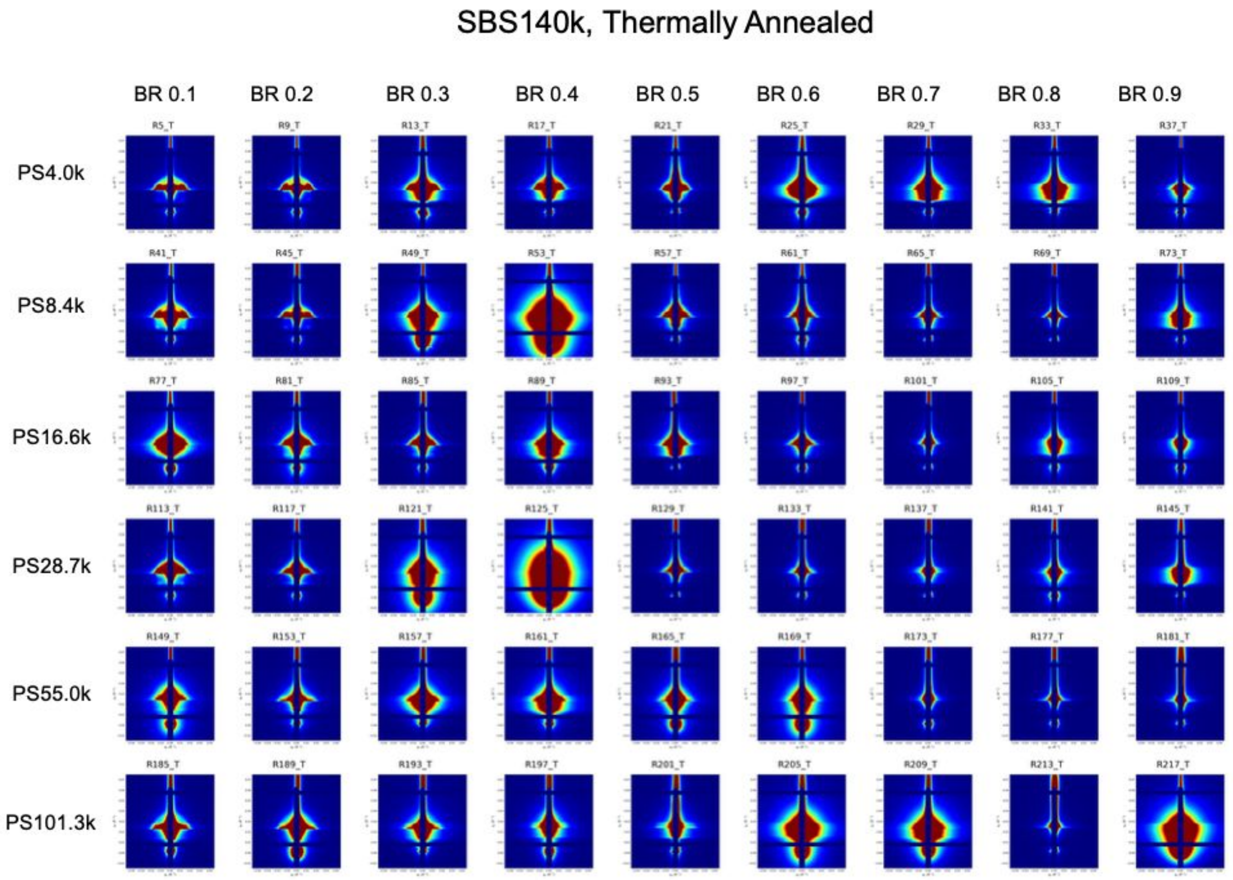

Figure S17: 2D scattering images for SBS140k/PS homopolymer with different mixing ratios for thermally annealed samples . Full resolution images are available in an online database.

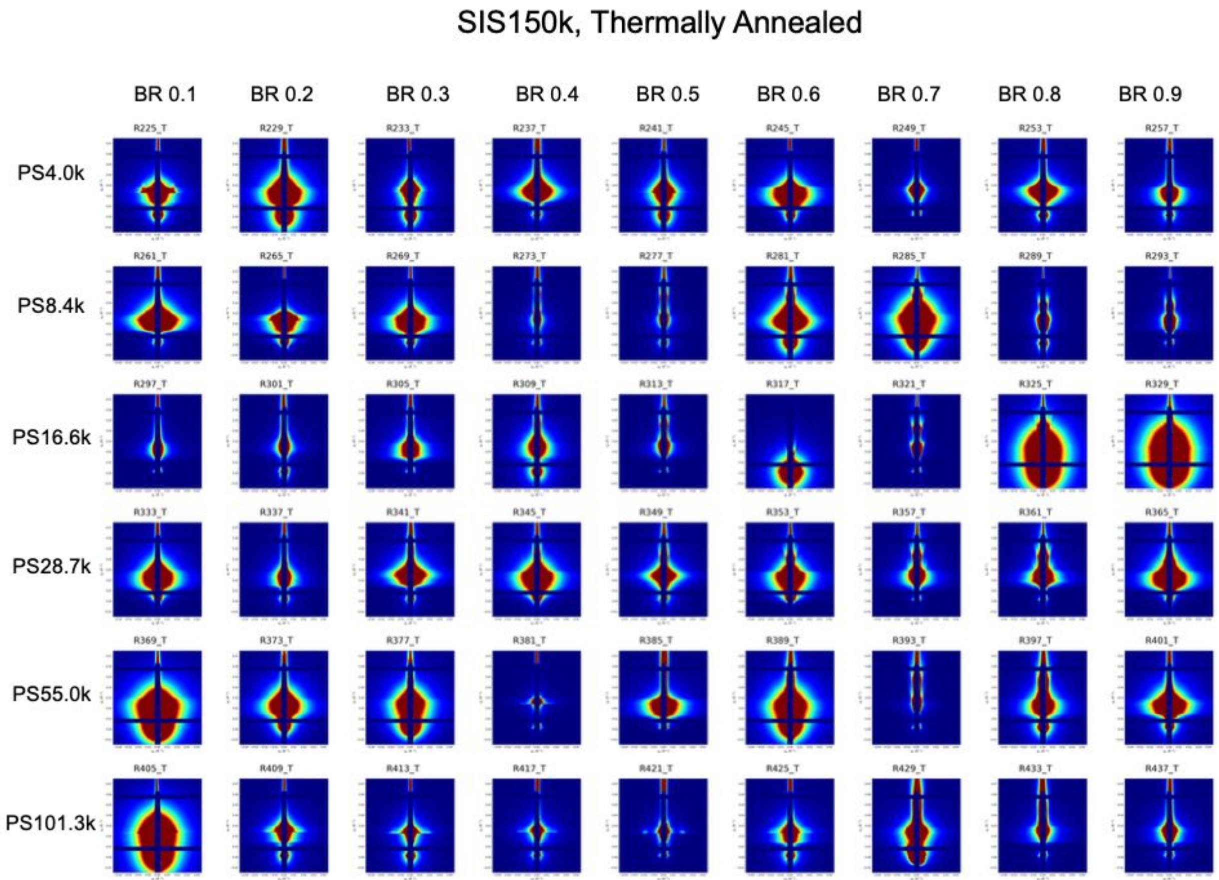

Figure S18: 2D scattering images for **SIS150k** with different mixing ratios for thermally annealed samples . Full resolution images are available in an online database.

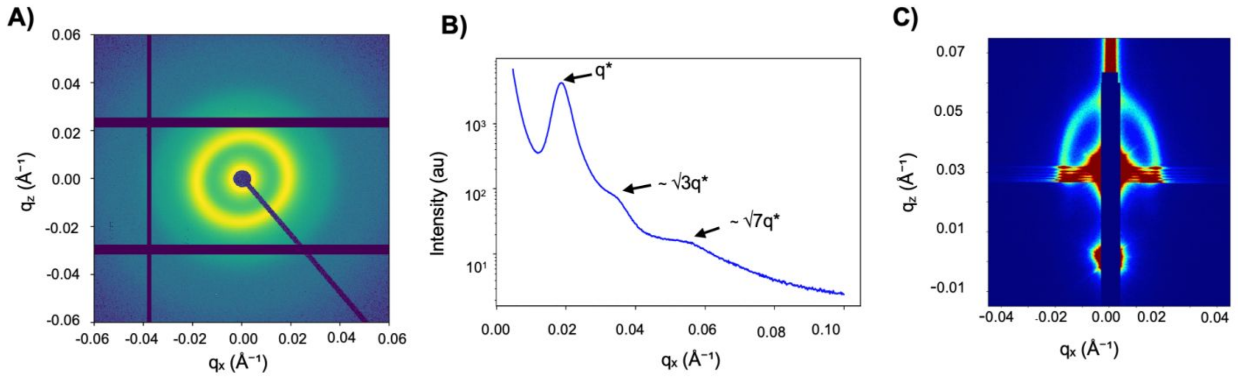

Figure S19: Native morphological characterization of the neat SBS (140 kDa) block copolymer. (A) 2D bulk small-angle X-ray scattering (SAXS) pattern of the neat SBS matrix. (B) Corresponding 1D bulk SAXS intensity profile exhibiting a strong primary scattering peak (q*) and a broad higher-order peaks positioned at √3q* and √7q*. (C) Representative 2D grazing-incidence small-angle X-ray scattering (GISAXS) pattern of the neat SBS thin film processed

under baseline additive annealing conditions (NVASA at a swell ratio of 3), confirming the retention of the microphase-separated structure in the rapidly processed thin-film state.

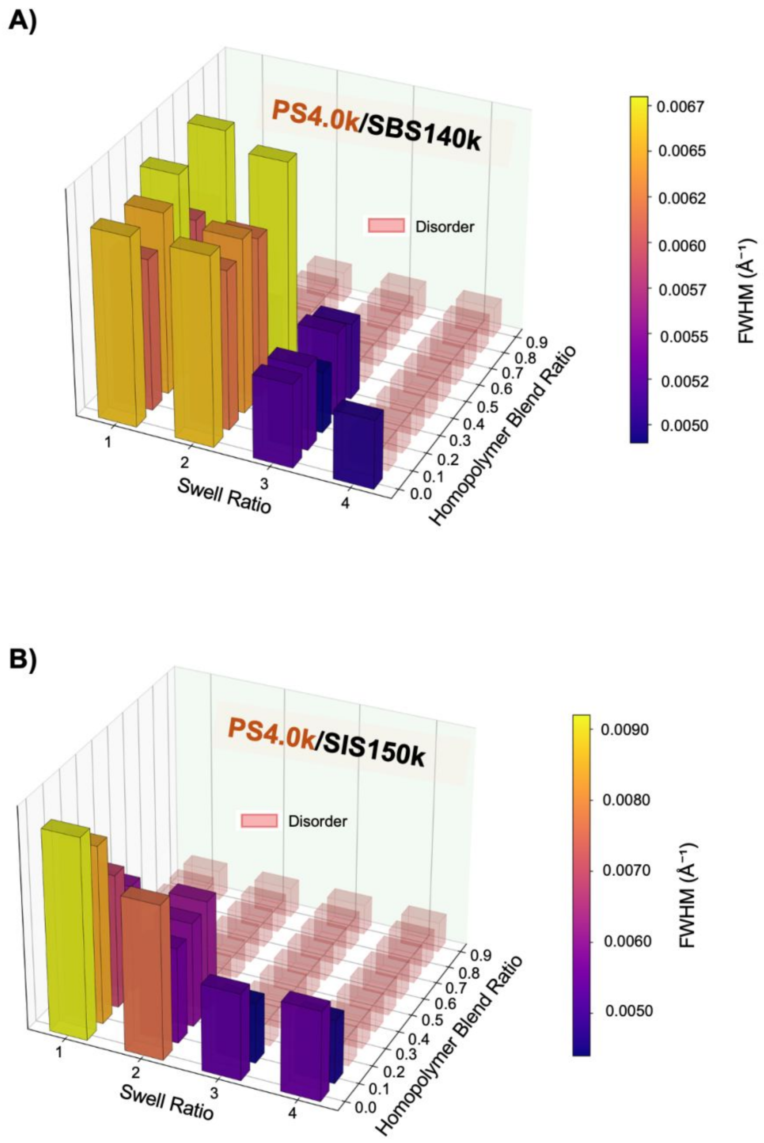

Figure S20: Evolution of the primary scattering peak Full Width at Half Maximum (FWHM) as a function of Swell Ratio (SR) and Homopolymer Blend Ratio. (A) 3D bar plot for the PS4.0k/SBS140k blend system. (B) 3D bar plot for the PS4.0k/SIS150k blend system. In both panels, the height and color of the solid bars correspond to the measured FWHM. Faded pink, transparent bars denote disordered states where a distinct primary peak is lost.

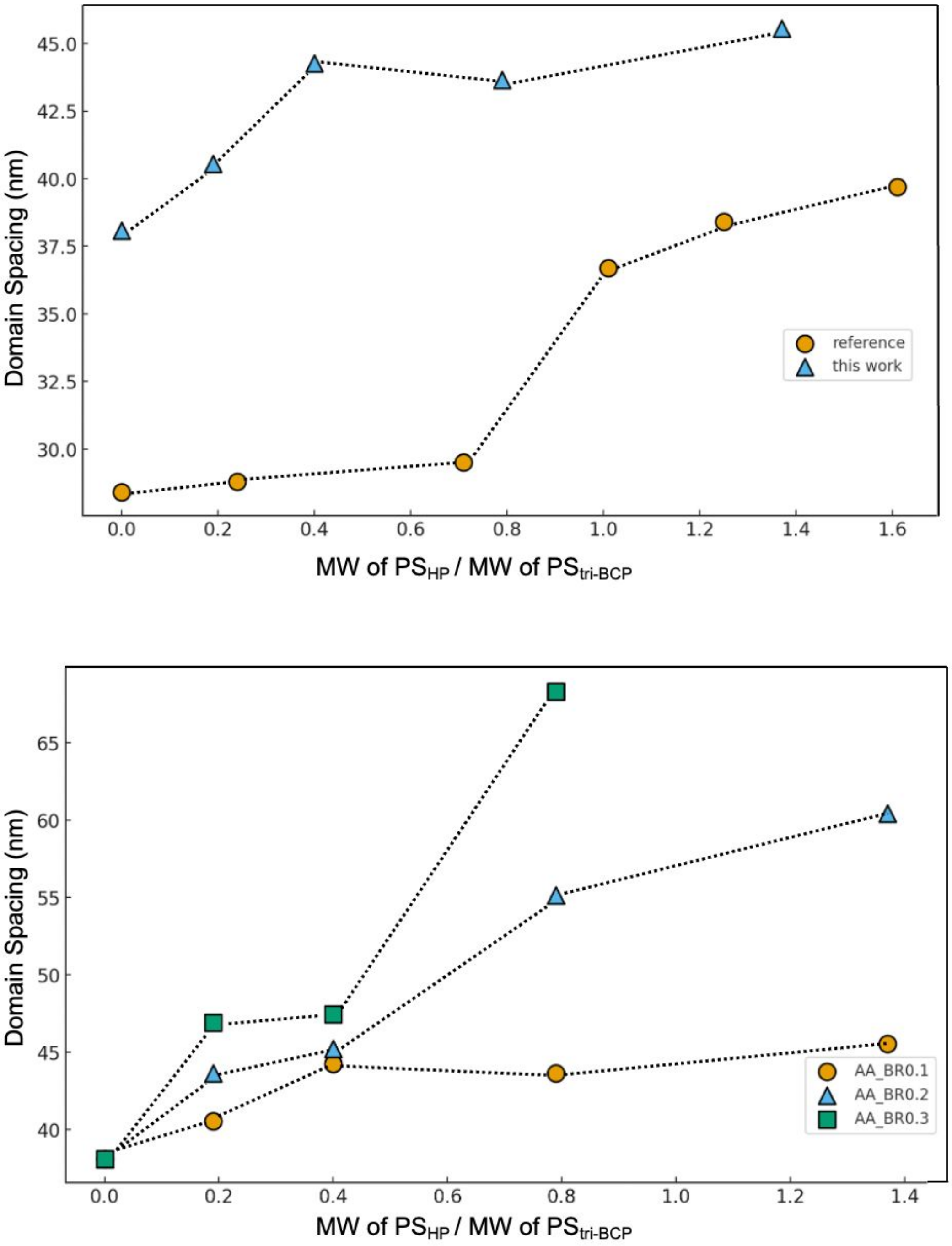

Figure S21: Comparison of domain spacing at 10 wt% blending of PS homopolymer of different MWs with SBS triblock copolymer between this work and reference work reported.[1]

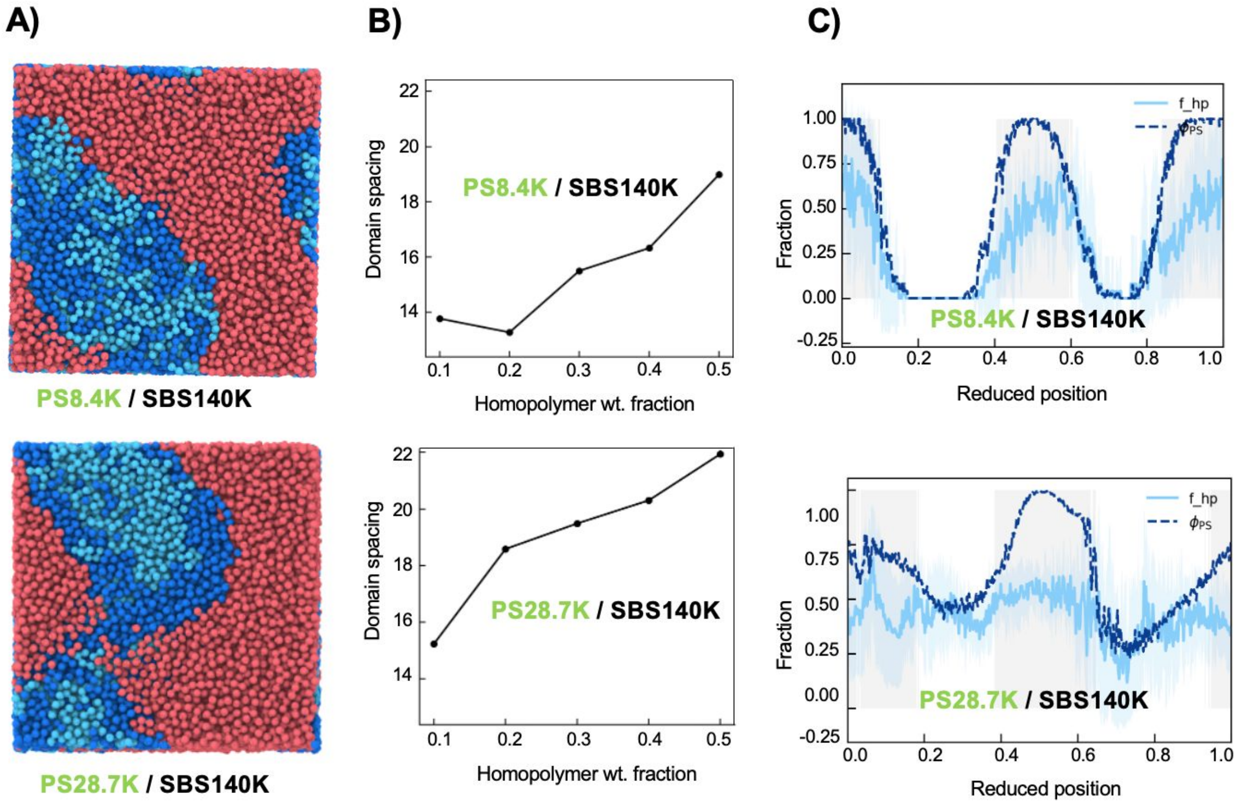

Figure S22. Coarse-grained molecular simulation results for PS/SBS triblock copolymer–homopolymer blends showing A) morphology evolution at homopolymer wt. fraction of 0.2, B) domain spacing evolution with increasing homopolymer wt. fraction, and C) folded composition profile at homopolymer wt. fraction of 0.2 . In the simulation setup, the light blue beads represent HP PS, while the dark blue beads represent PS in the triblock copolymer domain, and the red beads represent the butadiene domains

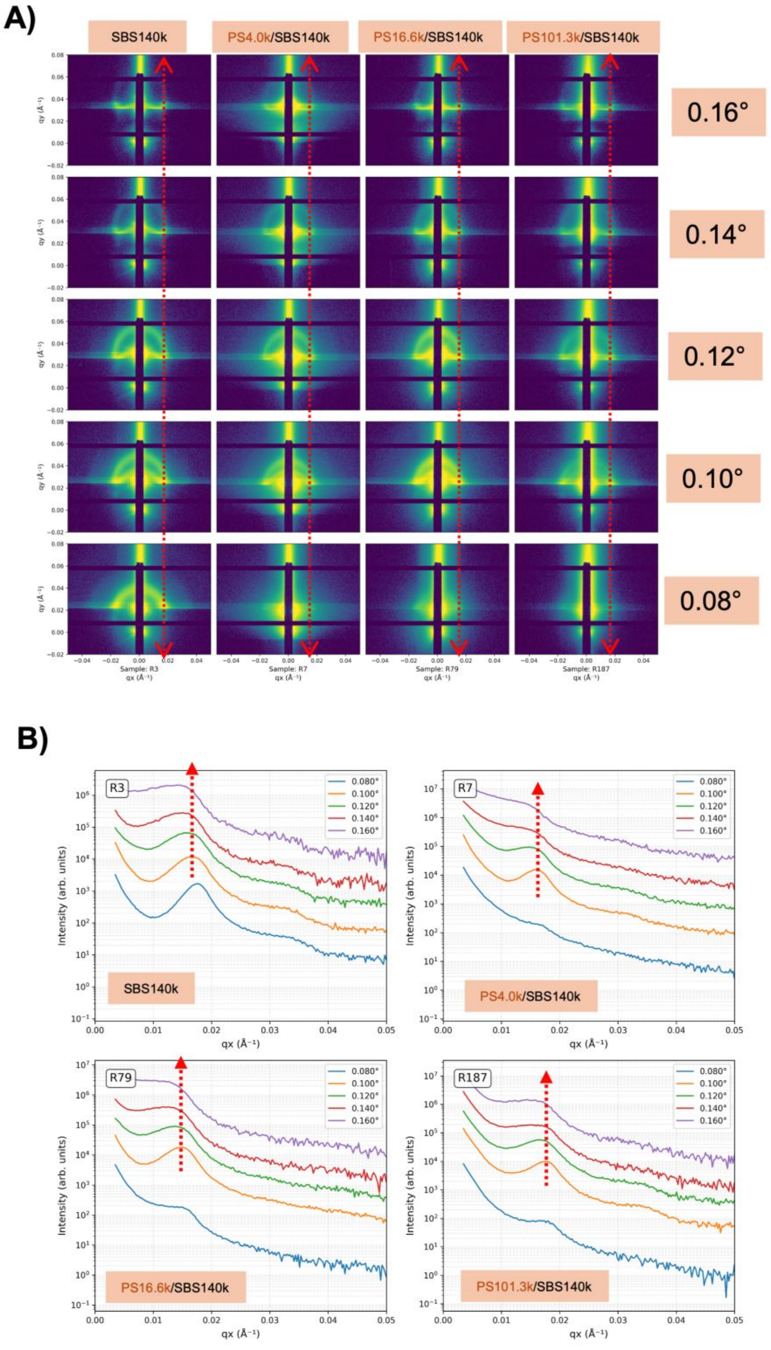

Figure S23. Depth-dependent GISAXS analysis confirming uniform bulk morphology across different blend regimes. **(A)** 2D scattering patterns and **(B)** corresponding 1D scattering profiles for the neat SBS140k triblock and representative blends with low, medium, and high MW homopolymers (BR = 0.1). Data were collected at incident angles ranging from 0.08° (surface-sensitive) to 0.16° (fully penetrating the film bulk). The vertical red dashed lines highlight the primary scattering peak. At incident angles of 0.10° and above, the primary scattering peaks remain highly consistent in both position and shape across all depths, confirming that the microphase-separated morphology exists uniformly throughout the film bulk and resolving apparent surface texturing observed via AFM.

Table S1: Comparison between NOVA (decoupled high-throughput architecture) with other similar existing robotic platforms (mostly integrated) reported.

Platform	Material Focus	Parameter Space	Throughput	Architecture<br>(In-situ Feasibility)
**NOVA**	Solid-state films	HP MW, Blend Ratio, Swell Ratio, Annealing Method	Film prep ~3 min/sample; GISAXS ~4–5 min/sample	**Decoupled**; in-situ unfeasible due to synchrotron requirements
**PolyBot**[2]	Electronic polymers	Ink formulation, Doping, Deposition, Heating	Continuous inline fabrication & measurement	**Integrated**; feasible with UV-Vis & electrical probes
**Ada**[3]	Spin-coated organic films	Ink composition, Spin speed, Annealing time	~20 min/sample	**Integrated but sequential**; bottleneck limits throughput
**PANDA**[4]	Electrodeposited films	Monomer conc., Deposition time, Voltage	Parallel sweep across 96-well plates	**Integrated**; feasible with optical/electrochemical probes
**AFL**[5]	Liquid soft materials	Liquid composition, Concentrations	Continuous inline flow-cell measurement	**Integrated**; limited by X-ray flux

Table S2: Theoretical calculation of χN values for each triblock copolymer system (first column). The N (degree of polymerization) values for each block (second and third column) calculated to get effective, total N (fourth column). χ values range (fifth column) at equilibrium temperatures, taken based on literature reports to calculate χN range (sixth column).

**Triblock BCP system**	N_{PS}	$N_{PB\ or\ PI}$	N_{total}	**χ range**	**χN range**
**PS-PB-PS** (21-b-98-b-21 kDa)	404	1815	2219	0.02 - 0.05 [6, 7]	44.38 – 110.95
**PS-PI-PS** (11.25-127.5-11.25 kDa)	216	1875	2091	0.03 - 0.06 [8, 9]	62.73 – 125.46

Table S3: Details of each triblock/homopolymer blend system (first column), the ratio value of PS MW between the PS of HP and PS of triblock(second column), weight fractions of homopolymer and their full width half maximum (FWHM), peak position and domain spacing values for corresponding systems (third, fourth, fifth and sixth column)

**HP/ Triblock BCP system**	**MW of $PS_{HP}/PS_{tri-BCP}$**	**HP Blend Ratio**	**FWHM ($Å^{-1}$)**	**Peak Position ($Å^{-1}$)**	**Domain Spacing (nm)**
**SBS140k**	0	-	0.0052	0.0165	38.09
**PS4.0k/SBS140k**	0.19	0.1	0.0052	0.0155	40.55
		0.2	0.0049	0.0144	43.65
		0.3	0.0052	0.0134	46.90
		0.4	0.0051	0.0125	50.28
		0.5	Disordered	Disordered	Disordered
**PS8.4k/SBS140k**	0.40	0.1	0.0058	0.0142	44.26
		0.2	0.0062	0.0138	45.22
		0.3	0.0067	0.0132	47.43
		0.4	Disordered	Disordered	Disordered
**PS16.6k/SBS140k**	0.79	0.1	0.0053	0.0144	43.65
		0.2	0.0065	0.0114	55.13
		0.3	0.0063	0.0092	68.32
		0.4	Disordered	Disordered	Disordered

PS28.7k/SBS140k	1.37	0.1	0.0044	0.0138	45.55
		0.2	0.0066	0.0104	60.44
		0.3	Disordered	Disordered	Disordered
PS55.0k/SBS140k	2.62	0.1	0.0055	0.0151	41.63
		0.2	0.0054	0.0158	39.78
		0.3	0.0063	0.0150	41.90
		0.4	0.0059	0.0152	41.35
		0.5	Disordered	Disordered	Disordered
PS101.3k/SBS140k	4.82	0.1	0.0049	0.0171	36.75
		0.2	0.0056	0.0161	39.04
		0.3	0.0059	0.0158	39.78
		0.4	0.0054	0.0152	41.35
		0.5	0.0054	0.0147	42.75
		0.6	Disordered	Disordered	Disordered
SIS150k	0	-	0.0053	0.0184	34.16
PS4.0k/SIS150k	0.35	0.1	0.0044	0.0144	43.65
		0.2	Disordered	Disordered	Disordered
PS8.4k/SIS150k	0.75	0.1	0.0043	0.0131	47.98
		0.2	Disordered	Disordered	Disordered
PS16.6k/SIS150k	1.47	0.1	0.0047	0.0132	47.61
		0.2	0.0055	0.0142	44.26
		0.3	0.0052	0.0142	44.26
		0.4	Disordered	Disordered	Disordered
PS28.7k/SIS150k	2.55	0.1	0.0051	0.0166	37.86
		0.2	0.0047	0.0168	37.41
		0.3	Disordered	Disordered	Disordered
PS55.0k/SIS150k	4.88	0.1	0.0043	0.0174	36.12
		0.2	0.0055	0.0173	36.33
		0.3	0.0055	0.0172	36.54
		0.4	0.0066	0.0163	38.56
		0.5	Disordered	Disordered	Disordered
PS101.3k/SIS150k	9.00	0.1	0.0044	0.0171	36.76
		0.2	0.0056	0.0169	37.19
		0.3	0.0057	0.0174	36.12
		0.4	Disordered	Disordered	Disordered

References

(1) Sik Park, D.; Sancaktar, E. Structural Parameters for Nanocylinder Microdomains of Polystyrene-Polybutadiene-Polystyrene Triblock Copolymer and Its Blends with Polystyrene Homopolymer. *Curr. Nanosci.* **2012**, *8*, 244–248.
(2) Wang, C.; Kim, Y.-J.; Vriza, A.; Batra, R.; Baskaran, A.; Shan, N.; Li, N.; Darancet, P.; Ward, L.; Liu, Y. Autonomous Platform for Solution Processing of Electronic Polymers. *Nat. Commun.* **2025**, *16*, 1498.
(3) MacLeod, B. P.; Parlane, F. G.; Morrissey, T. D.; Häse, F.; Roch, L. M.; Dettelbach, K. E.; Moreira, R.; Yunker, L. P.; Rooney, M. B.; Deeth, J. R. Self-Driving Laboratory for Accelerated Discovery of Thin-Film Materials. *Sci. Adv.* **2020**, *6*, eaaz8867.
(4) Quinn, H.; Robben, G. A.; Zheng, Z.; Gardner, A. L.; Werner, J. G.; Brown, K. A. PANDA: A Self-Driving Lab for Studying Electrodeposited Polymer Films. *Mater. Horiz.* **2024**, *11*, 5331–5340.
(5) Beaucage, P. A.; Martin, T. B. The Autonomous Formulation Laboratory: An Open Liquid Handling Platform for Formulation Discovery Using X-Ray and Neutron Scattering. *Chem. Mater.* **2023**, *35*, 846–852.
(6) Bates, F. S.; Fredrickson, G. H. Block Copolymer Thermodynamics: Theory and Experiment. *Annu. Rev. Phys. Chem.* **1990**, *41*, 525–557.
(7) Sakurai, S.; Mori, K.; Okawara, A.; Kimishima, K.; Hashimoto, T. Evaluation of Segmental Interaction by Small-Angle X-Ray Scattering Based on the Random-Phase Approximation for Asymmetric, Polydisperse Triblock Copolymers. *Macromolecules* **1992**, *25*, 2679–2691.
(8) Khandpur, A. K.; Foerster, S.; Bates, F. S.; Hamley, I. W.; Ryan, A. J.; Bras, W.; Almdal, K.; Mortensen, K. Polyisoprene-Polystyrene Diblock Copolymer Phase Diagram near the Order-Disorder Transition. *Macromolecules* **1995**, *28*, 8796–8806.
(9) Mori, K.; Hasegawa, H.; Hashimoto, T. Small-Angle X-Ray Scattering from Bulk Block Polymers in Disordered State. Estimation of χ-Values from Accidental Thermal Fluctuations. *Polym. J.* **1985**, *17*, 799–806.
